# Transcriptomics reveals multiple resistance mechanisms against cotton leaf curl disease in a naturally immune cotton species, *Gossypium arboreum*

**DOI:** 10.1038/s41598-017-15963-9

**Published:** 2017-11-21

**Authors:** Rubab Zahra Naqvi, Syed Shan-e-Ali Zaidi, Khalid Pervaiz Akhtar, Susan Strickler, Melkamu Woldemariam, Bharat Mishra, M. Shahid Mukhtar, Brian E. Scheffler, Jodi A. Scheffler, Georg Jander, Lukas A. Mueller, Muhammad Asif, Shahid Mansoor

**Affiliations:** 1 Agricultural Biotechnology Division, National Institute for Biotechnology and Genetic Engineering (NIBGE), Jhang Road, Faisalabad, Punjab Pakistan; 2 Pakistan Institute of Engineering & Applied Sciences (PIEAS), Nilore, Islamabad, Pakistan; 3 Boyce Thompson Institute, 533 Tower Road, Cornell University, Ithaca, NY USA; 4 Nuclear Institute for Agriculture & Biology (NIAB), Jhang Road, Faisalabad, Punjab Pakistan; 5 Department of Biology, University of Alabama at Birmingham, Birmingham, AL USA; 6 Genomics and Bioinformatics Research Unit (USDA-ARS), Stoneville, MS USA; 7 Crop Genetics Research Unit, United States Department of Agriculture-Agricultural Research Service (USDA-ARS), Stoneville, MS USA; 8 Present Address: AgroBioChem Department, Gembloux Agro-Bio Tech, University of Liège, 5030 Gembloux, Belgium

## Abstract

Cotton leaf curl disease (CLCuD), caused by cotton leaf curl viruses (CLCuVs), is among the most devastating diseases in cotton. While the widely cultivated cotton species *Gossypium hirsutum* is generally susceptible, the diploid species *G. arboreum* is a natural source for resistance against CLCuD. However, the influence of CLCuD on the *G. arboreum* transcriptome and the interaction of CLCuD with *G. arboreum* remains to be elucidated. Here we have used an RNA-Seq based study to analyze differential gene expression in *G. arboreum* under CLCuD infestation. *G. arboreum* plants were infested by graft inoculation using a CLCuD infected scion of *G. hirsutum*. CLCuD infested asymptomatic and symptomatic plants were analyzed with RNA-seq using an Illumina HiSeq. 2500. Data analysis revealed 1062 differentially expressed genes (DEGs) in *G. arboreum*. We selected 17 genes for qPCR to validate RNA-Seq data. We identified several genes involved in disease resistance and pathogen defense. Furthermore, a weighted gene co-expression network was constructed from the RNA-Seq dataset that indicated 50 hub genes, most of which are involved in transport processes and might have a role in the defense response of *G. arboreum* against CLCuD. This fundamental study will improve the understanding of virus-host interaction and identification of important genes involved in *G. arboreum* tolerance against CLCuD.

## Introduction

Cotton (*Gossypium spp.)* is a leading source of natural textile fiber, oil and protein meal, making it one of the most important cash crops in many countries^1^. The major cotton producing countries include China, India, United States, and Pakistan which contribute more than 70% of the total cotton production around the globe (https://apps.fas.usda.gov/). Among the 50 *Gossypium* species, two diploid species (*G. herbaceum and G. arboreum*) and two tetraploid species (*G. hirsutum* and *G. barbadense*) are cultivated worldwide, but *G. hirsutum* (upland cotton) accounts for more than 90% of overall cotton production^2^.

Tetraploid cotton, *G. hirsutum*, evolved about 1–1.5 million years ago (Mya) as a result of a polyploidization event with an A-genome species *G. arboreum* and a D-genome species *G. raimondii* the putative progenitors^3^. *G. hirsutum* cultivars have superior lint yield and produce very high-quality fiber, however, these tetraploid cultivars are often more susceptible to several abiotic and biotic stresses^4,5^. In contrast, diploid cotton species, particularly *G. arboreum*, are more tolerant to many biotic and abiotic stresses including CLCuD. *G. arboreum* is a valuable source for novel genes to enhance genetic diversity as well as for specific traits^6,7^.

*Bemisia tabaci*, commonly known as silverleaf whitefly, is one of the sap sucking pests that causes damage to more than 500 plant species including cotton. Among the list of sap-sucking insects that cause damage to a cotton crop, whitefly is responsible for more than 50% crop losses^8^. Whitefly serves as a vector for viruses causing cotton leaf curl disease (CLCuD), which is a major threat to cotton crops in several countries including Pakistan and India^9^. Viruses causing CLCuD, collectively referred to as cotton leaf curl viruses (CLCuVs), belong to the family *Geminiviridae* and the genus *Begomovirus*, which is the most important genera of this family^9^. Other members of this genus also infect important crops including tomato, beans, chillies, cucurbits, mungbean and other vegetables^10–12^. Plants infected with CLCuD show typical begomovirus infection symptoms such as leaf curling, vein yellowing, leaf enations and stunted growth^9,13^. CLCuD is among the most devastating of viral diseases, responsible for serious crop losses annually that negatively impact a country’s economy^14^.

As their defense system, plants mobilize a variety of intrinsic mechanisms to deal with pathogens and it is necessary to understand the mechanisms that plants use to cope with different stresses at the genetic level^15^. Abiotic and biotic stresses activate a relay network of plant gene expression mechanisms that lead to the reprogramming of a variety of physiological and metabolic processes in accordance with the stress response. Early studies mainly used model plant species to identify a wide spectrum of genes that are involved in different levels of metabolism, signal transduction, osmotic regulation and stress response. Microarray gene expression profiling has been widely used to study differential gene expression in plants under different stresses^16,17^. Advances in the field of transcriptomics has made RNA-Sequencing (RNA-Seq) a major “-omics” technology for studying global gene expression of plants under different stress conditions^18,19^. Several RNA-Seq studies have been done to reveal mechanisms in cotton under different biotic stresses. Transcriptome analysis of cotton flower buds infested with the cotton boll weevil highlighted the diversity of genes and pathways regulated by pest infestation, such as kinase cascades, transcription factors (TFs), and phytohormone-signaling pathways^20^. Significant changes in the expression of transcripts were found to be associated with sugar and amino acid metabolism in cotton following aphid and whitefly infestation^21^. Another recent transcriptomic study reported several genes for control of phloem-feeding pests^22^.

Viral infection initiates a complex interaction between the virus and the host. Understanding host responses during viral infection can help in the development of effective strategies for virus control. In recent years, RNA-Seq studies have been applied extensively to uncover the responses of plant hosts to viral infection^23–29^. However, the comprehensive molecular mechanisms underlying CLCuD-cotton interactions remain poorly defined.

Therefore, to elucidate the mechanisms involved in cotton defense against CLCuD, we aimed to identify important differentially expressed genes in cotton under CLCuD infestation and to elucidate the resistance mechanisms of the naturally immune cotton species *G. arboreum* at a molecular level. We infested *G. arboreum* with CLCuD by grafting, then performed RNA-Seq followed by data analysis, weighted gene co-expression network analysis (WGCNA), gene ontology enrichment and functional annotation.

## Results and Discussion

Cotton is an economically important crop and CLCuD causes drastic losses in crop production. Compared to normally susceptible tetraploid *G. hirsutum*, the diploid species *G. arboreum* is a natural source for resistance against CLCuD. Typically, whitefly is the vector that transmits CLCuD in tetraploid cotton, but *G. arboreum* remains unaffected when infected with viruliferous whiteflies. Recent studies have revealed evidence that *G. arboreum* plants support CLCuD replication and its long-distance spread only if CLCuD is transmitted by graft inoculation. In the presence of CLCuV/CLCuMB_Bur_, mild symptoms of CLCuD have been shown to be induced in *G. arboreum* by its graft inoculation with scions from CLCuD infested *G. hirsutum* plants^30,31^. Interestingly, only a few leaves of *G. arboreum* have been observed to develop symptoms of the disease following grafting. The nature of this resistance pattern remains unknown and might be correlated with gene expression levels or other indigenous immune mechanisms of *G. arboreum* ^30,32^. The present study aims to detect changes *G. arboreum* gene expression in response to CLCuD infestation by graft inoculation, even in the absence of severe symptoms. This study will help in understanding the mechanisms involved in resistance of *G. arboreum* to the begomoviruses causing CLCuD.

### Grafting and transcriptome sequencing

**Figure 1:**
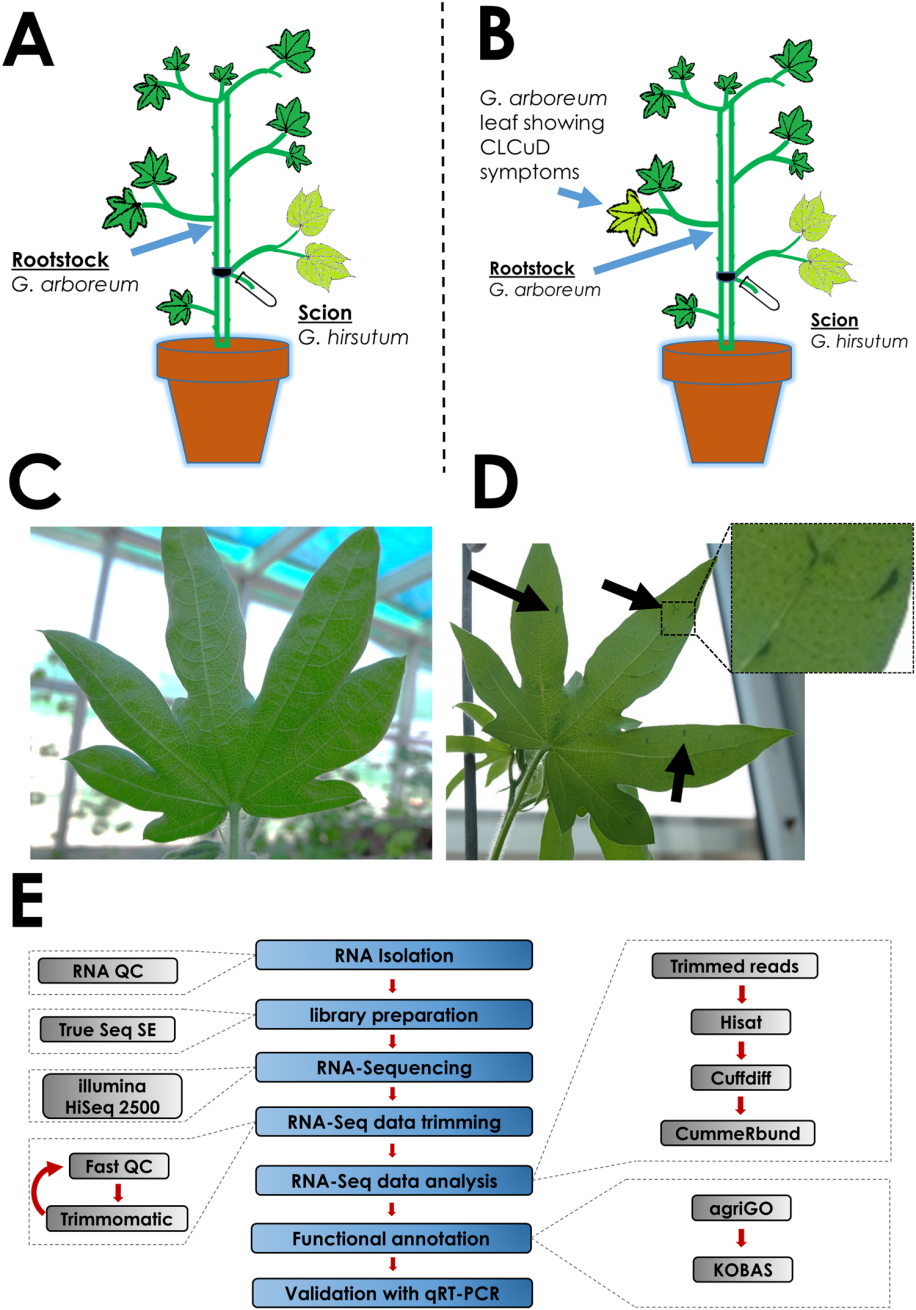
Experimental design, methodology and RNA-Seq pipeline used in study. Panel A and B show the graphical representation of grafting experiments (**A**) scion of cotton leaf curl disease (CLCuD) infected *Gossypium hirsutum* was used on a rootstock of *G arboreum*; asymptomatic leaves of CLCuD were collected for RNA extraction and sequencing. (**B**) Leaves showing symptoms of CLCuD were collected for RNA extraction and sequencing. (**C**) Shows an asymptomatic CLCuD free leaf of *G. arboreum*. (**D**) Shows a leaf with very mild symptoms of CLCuD including vein swelling and darkening, highlighted with black arrows. (**E**) Shows the workflow of the RNA-Seq experiment and the tools used at each step.

The standard method for CLCuD, delivery into *G. hirsutum* is by whitefly-mediated virus transmission. However, in most studies, *G. arboreum* remained asymptomatic and thought to be a non-host against CLCuD^33^. As an alternative, a graft infestation method was developed for systemic delivery of the CLCuD complex (cotton leaf curl geminivirus with its associated betasatellite) into *G. arboreum* ^30^. In this study, we used the graft-infestation method for delivery of CLCuD complex to *G. arboreum*. Characteristic viral infection symptoms were observed on *G. arboreum* grafted with CLCuD infected *G. hirsutum* scions (Fig. 1). Out of 30 plants grafted with scions, 27 plants established a successful graft. Among these 27 *G. arboreum* plants, 20 plants showed characteristic symptoms of CLCuD on the leaves near the grafted scion, while 7 plants remained asymptomatic for CLCuD (Fig. 1). These results are comparable with previous observations where most of the graft-infested *G. arboreum* developed mild symptoms with a few plants remaining asymptomatic^32^. RNA was extracted from three biological replicates of graft-infested, symptomatic and asymptomatic *G. arboreum* plants. This RNA was processed for library preparation followed by RNA sequencing.

### RNA-Seq data analysis

**Figure 2:**
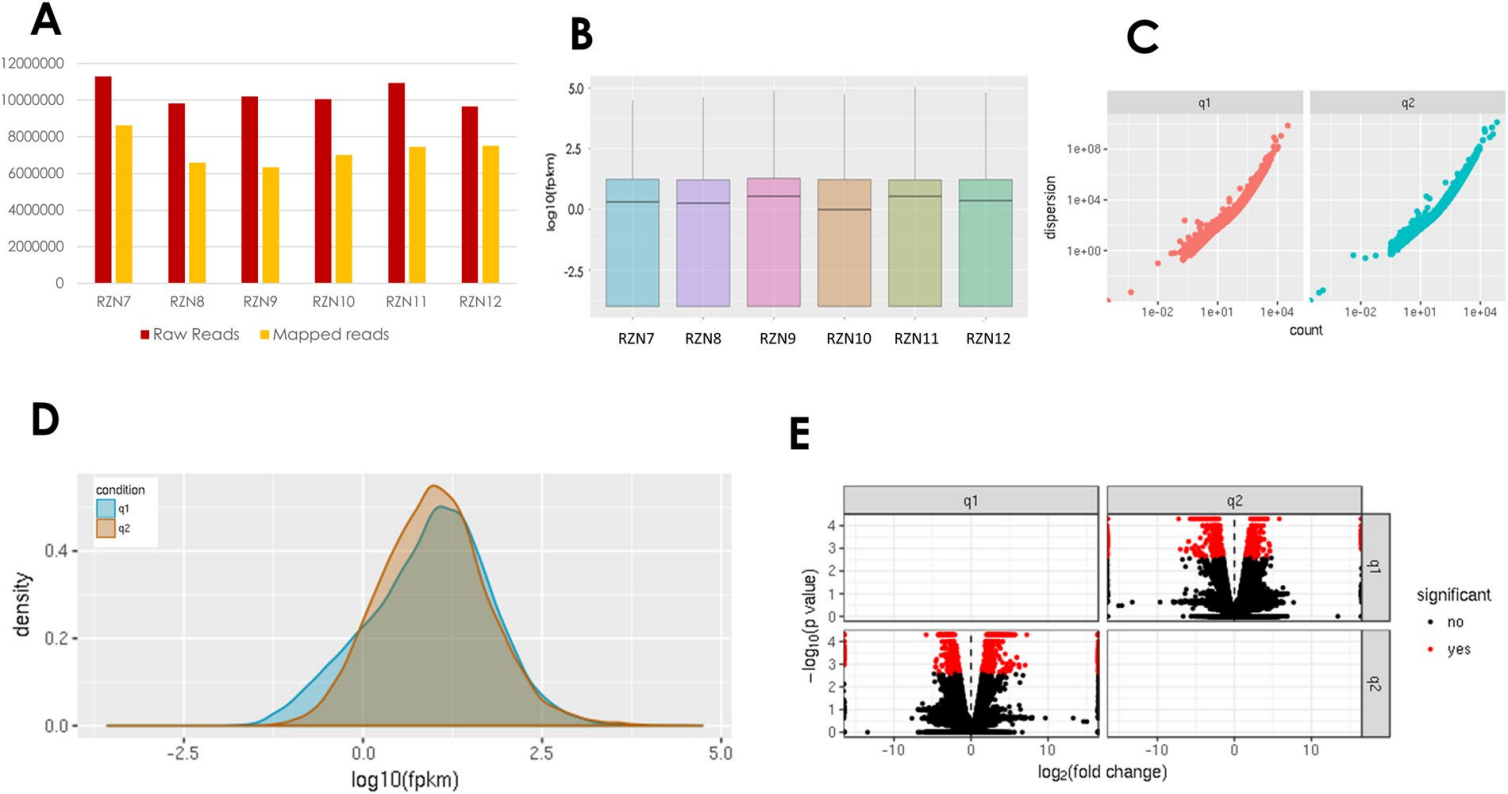
RNA-Seq data quality check and efficiency of mapping with *G. arboreum* genome. Panel A shows the raw sequencing reads compared the mapped reads where the y axis indicates the number of reads and x axis indicates the samples used in study. Panel B, C, D and E show the amount of data among replicates in terms of log10_(FPKM), gene dispersion, density and differentially expressed transcripts in the dataset. q1 and q2 represent the two conditions of symptomatic and asymptomatic *G. arboreum* respectively.

All six libraries were sequenced using a HiSeq™ 2500 platform. On average, 10 million total reads were obtained from each replicate (Table 1). Quality of individual sequences was evaluated using FastQC analysis, including per base sequence quality analysis which plots the Phred quality score distribution on the y axis for each read generated per sample and for each nucleotide base call plotted on the x axis (Figure S1). All FASTQ sequencing files obtained in this study have an average per base Phred score of 64, a conventional threshold denoting high quality base calls. High quality sequence reads were aligned to the *G. arboreum* reference genome with 13 chromosomes and 41,330 gene models (Table S1). The percentage of mapped reads obtained from the alignment summary of HISAT2 is provided in Table 1 (Fig. 2A).

**Table 1:** Summary of RNA-Seq runs used in this study.

Sample	Condition	No. of Reads	% Mapping
RZN7	Grafted - CLCuD symptomatic	11304210	76.25
RZN8	Grafted - CLCuD symptomatic	9833891	66.97
RZN9	Grafted - CLCuD symptomatic	10201621	62.17
RZN10	Grafted - asymptomatic	10049099	69.77
RZN11	Grafted - asymptomatic	10941016	68.2
RZN12	Grafted - asymptomatic	9667217	77.69

**Figure 3:**
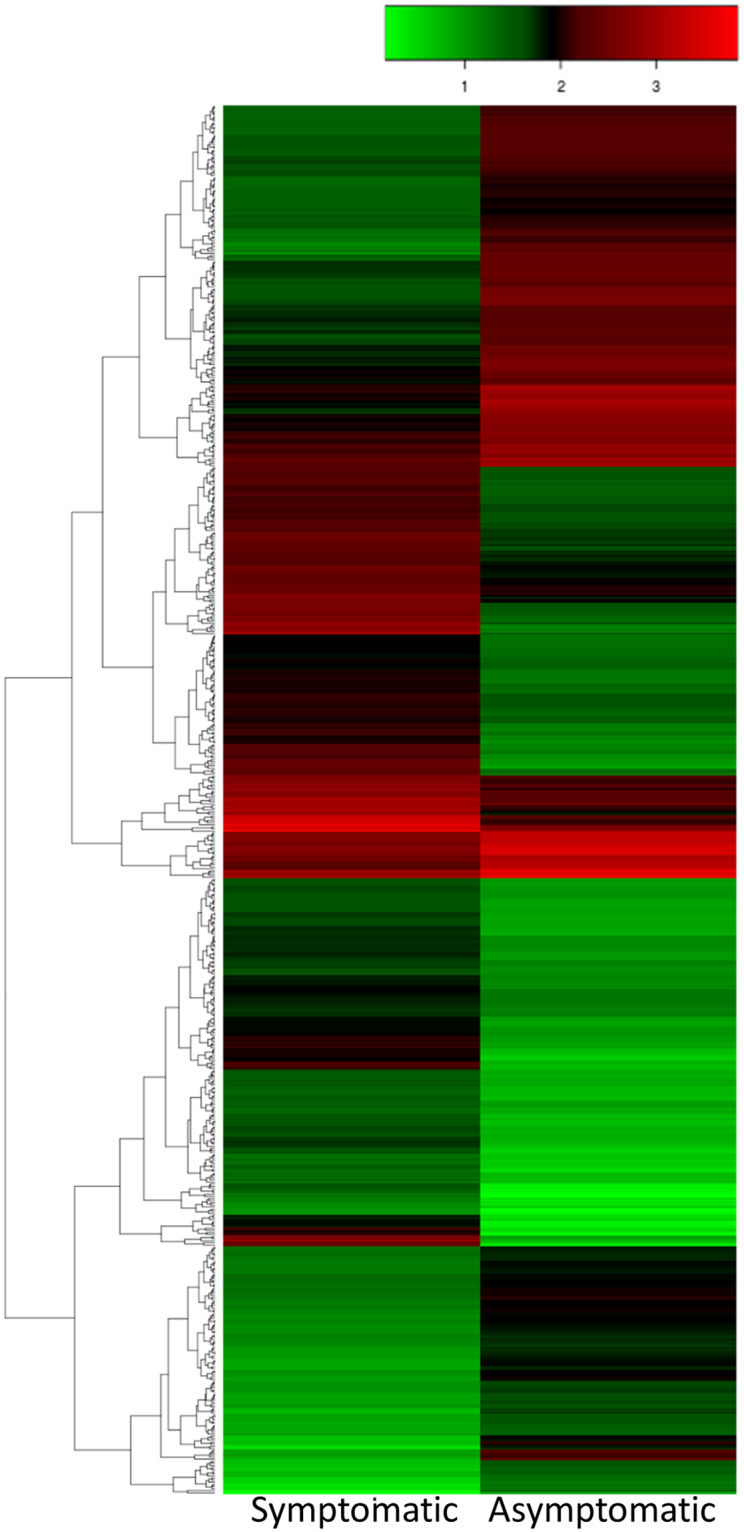
Hierarchical clustering of differentially expressed genes. A heat map of *G. arboreum* differentially expressed genes in response to cotton leaf curl disease with respect to hierarchical clustering. Log10 expression values were used for the analysis and negative values were set to zero. Clustering and the heat map were performed using heatmap 2.0 package in R.

Differential gene expression was quantified and grafted CLCuD symptomatic *G. arboreum* (RZN7-RZN9) were compared to the grafted asymptomatic *G. arboreum* (RZN10-RZN12). To assess the biological reproducibility of the experiment, each sample was treated as an individual biological replicate using the cuffdiff package of Cufflinks^34^. We compared individual runs from each condition and calculated the transcript abundances of each independent run (Fig. 2B). A quality check of compared transcripts provided standard gene density and dispersion (Fig. 2C,D). The expression levels of mapped genes were normalized with a FPKM (fragments per kilobase of exon per million fragments mapped) value. To confirm the quality of RNA-Seq, the eight highest-ranking housekeeping control genes in *G. arboreum* were selected to evaluate gene expression^35^. Based on comparisons of our samples, none of these reference genes were significantly differentially expressed (Table 2), suggesting that the sequences obtained and the transcript expression levels qualified for further transcriptome analysis. In the comparison of gene expression levels of symptomatic and asymptomatic *G. arboreum* plants, an absolute value of log2 fold change >1 and the False Discovery Rate (FDR) <0.05 was set to declare differentially expressed genes (DEGs) involved in the response of CLCuD infestation. Overall, 1062 genes were differentially expressed in this comparison, out of which 563 and 499 were up and downregulated, respectively (Fig. 2E; Fig. 3).

**Table 2:** Expression levels of housekeeping control genes in *G. arboretum*.

No	Function	Gene_Id	log2FC	significant
1	Clathrin adaptor complexes medium subunit family protein	Cotton_A_09164_BGI-A2_v1.0	−0.363142	no
2	Catalytic subunit of protein phosphatase 2 A	Cotton_A_09192_BGI-A2_v1.0	0.619919	no
3	F-box family	Cotton_A_12507_BGI-A2_v1.0	0.510816	no
4	Betatubulin	Cotton_A_14308_BGI-A2_v1.0	−0.7817	no
5	Elongation factor	Cotton_A_23419_BGI-A2_v1.0	−0.452078	no
6	Glyceraldehyde-3-phosphate dehydrogenase C-2	Cotton_A_31637_BGI-A2_v1.0	−0.539749	no
7	Ubiquitin	Cotton_A_32873_BGI-A2_v1.0	0.0623818	no
8	Actin	Cotton_A_38366_BGI-A2_v1.0	−0.395614	no

The transcriptome data of all replicates from this experiment is publicly available under BioProject accession number PRJNA380937 and a comprehensive list of up- and downregulated DEGs is provided in Table S2. This data is a valuable resource for understanding CLCuD tolerance in *G. arboreum*. Furthermore, the DEGs identified were narrowed down by log2 fold-change, p values and q values for further experimentation. Many functional classes of genes related to pathogen defense were found to be significantly affected in CLCuD-infected symptomatic plants compared to that of the asymptomatic plants.

**Figure 4:**
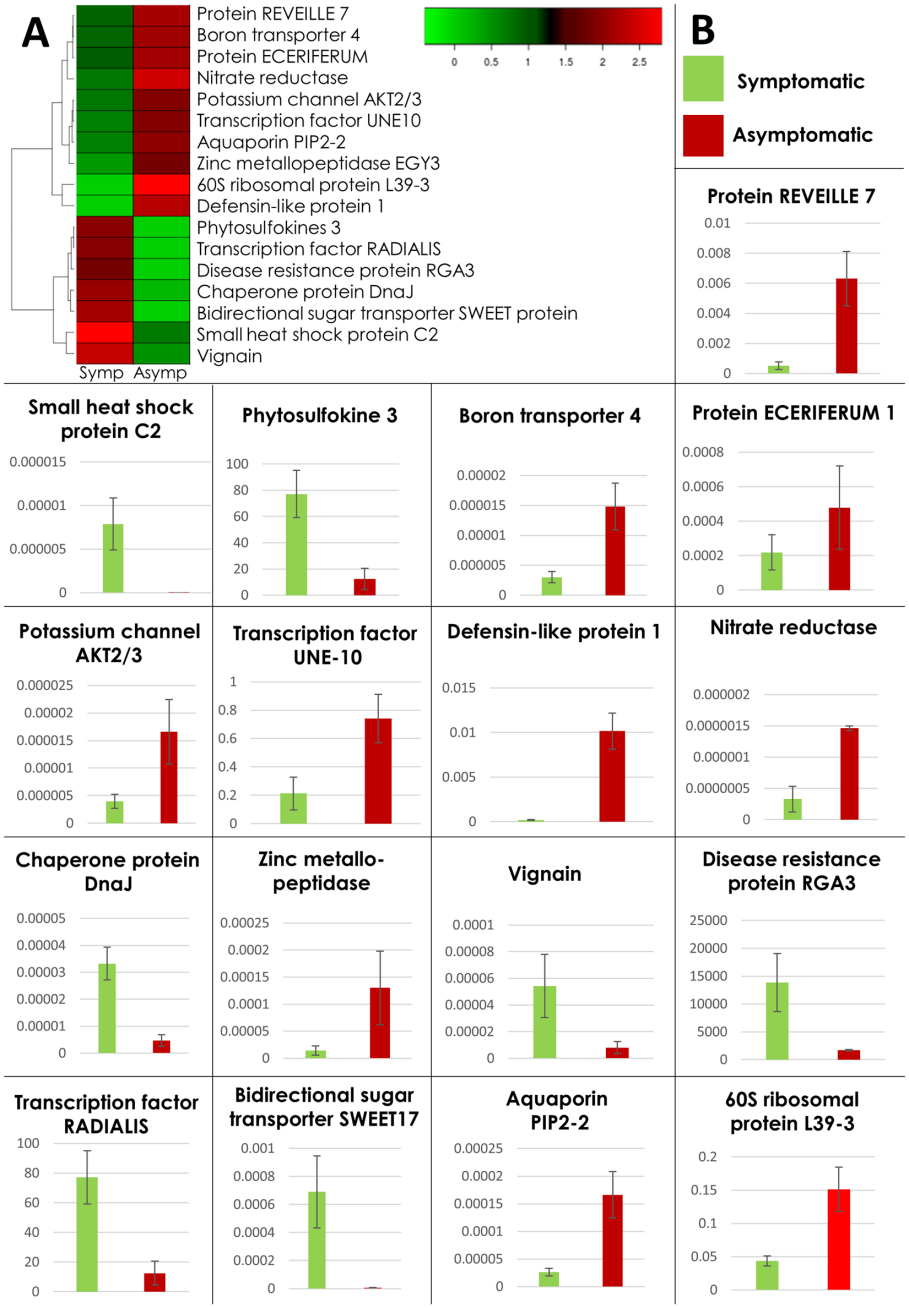
Validation of gene expression with quantitative RT-PCR. (**A**) Heat map of 17 selected DEGs for qRT-PCR in *G. arboreum* in response to cotton leaf curl disease with respect to hierarchical clustering. Log10 expression values were used for the analysis and negative values were set to zero. Clustering and the heat map were performed using heatmap 2.0 package in R. (**B**) Quantitative RT-PCR was used to measure the relative expression levels of seventeen pathogen resistance related genes with 18 S as an internal reference. Values were expressed as fold changes of transcript levels in the CLCuD infested symptomatic leaf samples with respect to the transcript levels in CLCuD infested asymptomatic leaf samples. Error bars represented standard error (SE) of three biological replicates.

For the validation of our transcriptomic data we selected 17 significant DEGs with a probable role in disease resistance. We designed primers and performed qPCR on cDNA of independent biological replicates of *G. arboreum*. Our qPCR data strongly correlated with the RNA-Seq expression data (Fig. 4). The implication of these genes has been discussed with reference to published data (Supplementary Discussion; Table 3).

**Table 3:** Selected differentially expressed genes for qRT-PCR and their probable functions in plant pathogen defense.

Gene	Probable role in defense	References
Probable zinc metallopeptidase EGY3	Development and stress response	^94^
Defensin-like protein 1	Bacterial and fungal pathogens as well as herbivorous insects	^95–97^
Phytosulfokines 3	Pattern-triggered immunity against pathogens, Leucine Rich Repeat family (LRR)	^98–100^
Chaperone protein DnaJ	Pathogen defense, antiviral defense	^101–103^
Transcription factor UNE10	Antiviral defense	^46^
Protein REVEILLE 7	Plant growth, stress, pathogen response	^104,105^
Small heat shock protein C2	Antiviral and antibacterial stress response	^59,106^
Transcription factor RADIALIS	Myb encoding genes, plant defense response	^45,107^
Putative disease resistance protein RGA3	R-gene mediated pathogen and disease response	^49,108,109^
Bidirectional sugar transporter SWEET17	Abiotic stress tolerance, pathogenesis related protein	^39^
Protein ECERIFERUM 1	Biotic and abiotic stresses	^110^
Probable aquaporin PIP2-2	Biotic and abiotic stresses, plant immunity	^111–113^
Potassium channel AKT2/3	Plant development, stress responses, antiviral defense	^41,42^
60 S ribosomal protein L39-3	Pathogen and disease resistance, antiviral defense	^7,114,115^
Boron transporter 4	R-gene mediated viral defense	^38^
Vignain	Plant immunity, pathogenesis and plant defense	^116,117^
Nitrate reductase [NADH]	Pathogen signal-induced NO production	^118–120^

### Putative defense related genes involved in resistance of *G. arboreum* to CLCuD infection

Plants have evolved with sophisticated and well-established defense mechanisms to cope with pathogens, such as insects, fungi, bacteria and viruses^36^. Besides physical barriers (like trichomes), toxic compounds or secondary metabolites, plants have numerous defense pathways to defend themselves against diverse pathogens^37^. An initial gene ontology (GO) term analysis of the top one hundred most significantly expressed genes present in the RNA-Seq dataset indicated that several GO terms were involved in regulation of responses related to stress, external stimulus, biotic stress and plant defense (Figure S2).

Further dissection of expression levels of individual genes in symptomatic and asymptomatic *G. arboreum* plants revealed that many groups of genes were differentially expressed in response to CLCuD infection.

### Differential expression of membrane transporters and channel proteins

Among the DEGs, some transporter genes were observed such as a boron transporter gene that was upregulated while a SWEET 17 gene was downregulated in *G. arboreum* asymptomatic plants. An interaction of host boron transporter with viral coat protein can regulate boron transport to induce necrosis, or the interaction can modulate an R gene mediated defense response^38^. Based on these studies, the upregulation of the boron transporter gene in *G. arboreum* depicts the protection of the plant from boron toxicity. In *G. arboreum*, the boron transporter may also be involved in host-viral interaction which modulates the R-gene mediated response in the host to combat virus infection. SWEETs are bidirectional vacuolar fructose transporters in plants predicted to be involved in maintaining sugar homeostasis in plant organs during favorable as well as abiotic and biotic stress conditions. SWEET transporters have been found to be associated with plant pathogen interactions during a pathogen attack in plants^39^. A reduced level of *SWEET* gene expression in tobacco and rice was associated with enhanced resistance to pathogens, suggesting a role in pathogen growth and plant disease resistance^39,40^. The downregulation of *G. arboreum* SWEET transporter suggests its role in sugar homeostasis under unfavorable conditions and possible involvement in reduced pathogen growth and disease resistance.

Alteration in expression levels of channel proteins in plants has been reported as a response to environmental stimuli^41^. Significantly enhanced soybean mosaic virus resistance was observed in soybean by the overexpression of GmAKT2^42^. The induction of aquaporin channel proteins encoding genes has been reported with expression profiling of soybean under *Pseudomonas syringae* infection^43^. Studies have also shown the involvement of aquaporins in plant-viral interactions. For example, aquaporin genes interact with a cucumber mosaic virus (CMV) replication protein that potentially affects CMV replication in the host plant^44^. Thus, the upregulation of *G. arboreum* AKT2 and aquaporin genes might have a role in CLCuD.

### Transcription factors and R-gene mediated response

During pathogen attack, transcription factors play an important role in plant innate immunity. MYB transcription factors act as master regulators of cellular responses and are involved in plant development, secondary metabolism, hormone signal transduction, abiotic stress tolerance and disease resistance. These transcription factors have been found to be involved in mechanisms of disease resistance in several plants through regulation of defense genes^45^. In the present data set, differential gene expression of different MYB transcription factors, including RADIALIS and REVEILLE, indicate their role in plant defense against CLCuD. A recent study on genome-wide analysis of bHLH transcription factors in *Solanum lycopersicum* shows that bHLH transcription factors are involved in the plant’s defense under infection by T*omato yellow leaf curl virus* and upregulation of bHLH is associated with disease resistance^46^. *G. arboreum* bHLH transcription factor UNE10 was upregulated during CLCuD stress and suggests a role in disease resistance. Transcription factors, particularly ethylene response factor (ERF), lead to the transcriptional regulation of several jasmonate and ethylene responsive defense genes under pathogen attack^47^. In our experiment, seven DEGS were found to be ethylene responsive genes (Table S5) that might have a role in *G. arboreum* defense against CLCuD.

R gene-triggered resistance in plants is associated with a rapid defense response known as hypersensitive response (HR) that can bring localized cell death at the site of infection and trigger a series of downstream defense pathways^48^. R-genes have been found to be induced in response to several pathogens and diseases in plants, including tobacco mosaic virus, rice blast, Arabidopsis downy mildew, tomato leaf mold and Verticillium wilt-resistance in cotton^49^. In our experiment, eleven putative R genes were differentially expressed indicating their role in plant defense against CLCuD (Table S6). Protein kinases along with R genes are well known in disease resistance both in a positive and a negative manner^50,51^. Forty-six kinase genes were identified as DEGs and most of them were downregulated in asymptomatic *G. arboreum* (Table S7).

### Phytohormone signaling

CLCuD infestation in *G. arboreum* also revealed the induced expression of genes associated with phytohormone signaling pathways. Phytohormones are the key players in signaling cascades induced by pathogens^20^. In this study auxin, cytokinin, abscisic acid and brassinosteroid related genes were upregulated while genes involved in ethylene and salicylic acid pathways were downregulated, highlighting their role in plant defense. Taken together the complex cascade in phytohormone related gene expression demonstrates the interconnection of the signaling pathways regulated by phytohormones in response to CLCuD infection.

### Protein processing in endoplasmic reticulum and ubiquitin proteasome systems are involved in plant-viral interaction

Under virus infection, the plant’s cellular machinery is hijacked by viruses for DNA replication of viral genomes and translation of viral proteins. Like other pathogens including bacteria, phytopathogenic viruses activate endoplasmic reticulum (ER) stress signaling machinery. Viruses act as “pirates” and use host machinery for their spread across the plasmodesmata to neighboring cells using viral movement proteins (MP). The ER signaling mechanism has been found to be involved in cell to cell movement and spread of viruses through plasmodesmata^52^. In Arabidopsis, a broad range of viruses induce gene expression of ER stress marker genes such as Calreticulins (CRTs). Calreticulin has been shown to interact with movement of viral proteins including MP of tobacco mosaic and turnip crinkle virus (TCV). CRT helps in redirecting virus movement through plasmodesmata which leads to symptom development, however, its overexpression leads to delayed cell to cell movement of virus^53^. Many other well studied host proteins interact with viral MPs for regulation of their cell to cell movement including β−1,3-glucanase-interacting proteins^54^. DnaJ and heat shock chaperones act as potential translocation factors and are involved in regulation of viral cell-to-cell movement^55^. Deposition of β−1,3-glucan or callose in plasmodesmata initiates a hypersensitivity response (HR) in hosts, consequently leading to the blocking of plasmodesmata to hinder cell to cell viral movement. Viral proteins like the TGB2 protein of *Potato Virus X* (PVX) interacts with the host ß−1,3-glucanase to degrade the callose, followed by PVX movement through the plasmodesmata^54^. DnaJ (HSP 40) is an important co-chaperon of HSP70 and is important for its function^56^. HSP70 is proposed to be associated with coat protein interaction, viral transportation and replication of many geminiviruses. Downregulation of HSP70 showed decreased viral load and reduced viral movement in plants infected with tomato yellow leaf curl virus (TYLCV)^57^.

In *G. arboreum* symptomatic plants, the induction of many genes involved in protein processing in ER including CRT, lectin like gene OS9, glucanase interacting gene OST and heat shock proteins HSP70 and HSP 40 indicate the possible role of this pathway interacting with CLCuV proteins and helping viral cell to cell movement in the plant, making it susceptible to establishment of mild symptoms from graft inoculation with CLCuD. The downregulation of these genes in asymptomatic plants might have role in impairment of viral movement proteins, restricting viral movement in plasmodesmata (Figure S3).

The ubiquitin proteasome system (UPS) has a very important role in virus host interactions and plant defense against viruses^58,59^. There are several reports showing the complex interaction of host plant UPS components with viral proteins, suggesting that the ubiquitin pathway is probably a conserved pathway involved in plant virus interaction. UPS in plants is comprised of ubiquitin-activating enzyme (E1), ubiquitin-conjugating enzyme (E2), and ubiquitin-ligase (E3). These three enzymes make an E3 ubiquitin ligase complex which is required for polyubiquitination of cellular proteins, followed by 26 S proteasome targeted degradation^60^. During viral infection, the virus takes over the plant host’s UPS machinery regulating its gene expression to enhance pathogenesis, while in parallel the plant uses UPS as a strong layer of defense, mainly by targeting of viral proteins. Different components of UPS, from ubiquitin to 26 S proteasome have been found to be involved in plant defense mechanisms. Host UPS machinery is involved in targeted degradation of TMV and Turnip yellow mosaic virus movement proteins and decreased viral pathogenesis suggest a role for UPS in plant antiviral defense^59^.

Like other plant viruses, geminiviruses also interact with the different components of host UPS. Rep, a replication protein of geminiviruses, interacts with host plant SUMO-conjugating enzyme 1 (SCE1), which is an essential host factor for viral infection in the plant^61^. Altered expression of SCE1 leads to unsuccessful geminiviral replication and suppression of infection^62^. SKP1 is another potent component of the CUL1-based SCF ubiquitin E3 ligases that recruits proteins for polyubiquitination. Geminiviral C2 protein interferes with the different components of host UPS including SKP1^59,63^. Suppression of SKP1 in plants abolishes the downstream HR and resistance response in plants^60^. βC1 from *Cotton leaf curl Multan virus* (CLCuMV) interacts with the host ubiquitin-conjugating (UBC) enzyme, SlUBC3, leading to symptom induction that is possibly due to downregulation of the UPS^64^. The upregulation of UPS pathway components including UBC3 (COP1), SCE1(UBE21) and SKP1 in the *G. arboreum* asymptomatic plants indicates that ubiquitin mediated proteolysis could be a defense response against symptom development of CLCuD (Figure S4).

### Weighted co-expression gene network analysis (WGCNA) identifies two novel modules within the network

**Figure 5:**
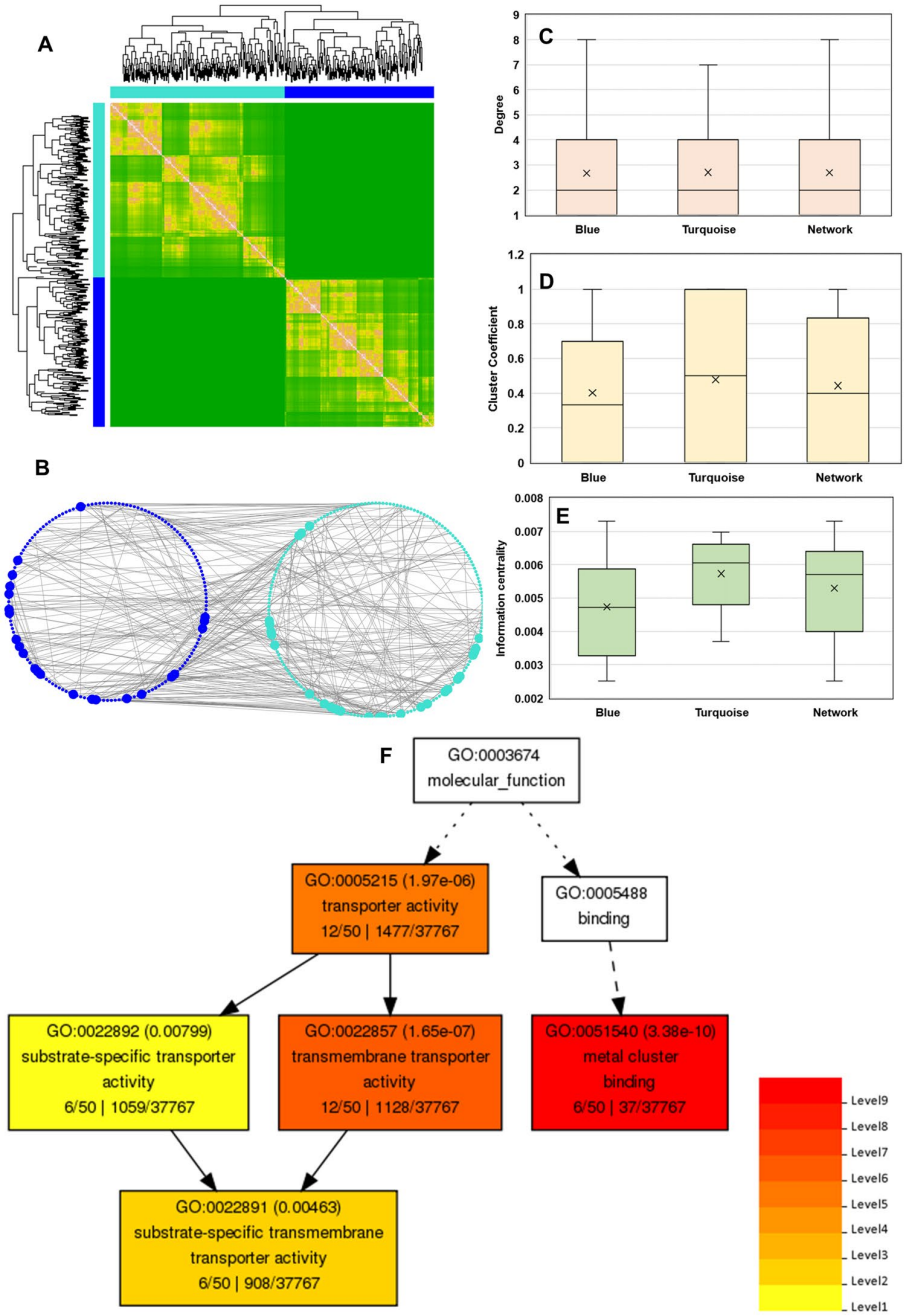
Weighted gene co-expression network construction and analysis for CLCuD-responsive cotton genes in CLCuD symptomatic and asymptomatic leaves. (**A**) Transcriptomic data from symptomatic and asymptomatic leaves was analyzed by WGCNA using 468 DEGs. Genes were clustered as per expression arrangements characterized by the dendrogram and topological overlap mapping metric (TOM) heat map. Each line of the dendrogram corresponds to a gene. Clusters of similarly-regulated genes are grouped as modules by corresponding color (blue and turquoise) with a threshold minimum module size of 70 genes. The intensity of pink coloring in heatmap specifies high strength and green as no strength of correlation between pairs of genes on a direct scale and features hierarchical clustering dendrograms possessing a range of weighted correlations. (**B**) Weighted network illustrates correlations (edges) among the nodes (genes) with a weighted correlation threshold of ≥0.85. The network is composed of 468 connections and 348 genes organized in two different modules. The node color corresponds to modules identified *via* WGCNA. Nodes with high connectivity ( ≥5 connections, hubs) among different modules are indicated with increased node size. (**C**) Highly connected nodes (hubs) within the co-expression network are shown. Average degree of turquoise and blue modules is not changed in the entire co-expression network (2.689655172). (**D**) Clustering coefficient (degree to which a node is connected in a neighborhood) for genes within each node is illustrated in a box plot. Turquoise module displays significantly a higher average clustering coefficient than whole co-expression network (0.443746579). (**E**) The information centrality (the flow of information between any two nodes in a connected network) for the largest component of co-expression network (92 nodes) is presented. Turquoise module displays significantly increased information centrality compared to the entire network (0.005299235). (**F**) Interactive graph of GO terms associated with cotton leaf curl disease responsive *G. arboreum* hub genes identified by co-expression network analysis. Analysis performed with online tool agriGO (bioinfo.cau.edu.cn/agriGO/) where a key indicates significance levels of GO terms.

With the availability of large-scale transcriptome datasets, co-expression network analysis allows identification of a cohort of genes with similar expression patterns in response to a given stimulus or physiological condition within a cell^65,66^. Thus, co-expression networks can identify a set of genes, which might participate in a common biological process. To determine CLCuD-responsive common gene signatures, we performed a weighted gene co-expression network analysis^67,68^ on 468 selected DEGs. Using the WGCNA platform, we created a topological overlap mapping metric (TOM) plot, a measure of neighborhood proximity that calculates the similarity matrix of gene expression between two nodes^69^. TOM also features hierarchical clustering dendrograms possessing a range of weighted correlations^67^. These analyses led us to generate an undirected weighted network with scale-free topology, a network with power-law degree distribution. This weighted co-expression network encompasses two different modules (Fig. 5A,B; Table S8) that are denoted with two different colors. The turquoise and blue modules comprise of 252 and 216 genes, respectively.

Centrality measures can reveal the most influential vertices in a network. To decipher the most important nodes within this co-expression network, we calculated, degree (number of connections of a node) and clustering coefficients (degree to which a node is connected in a neighborhood) as well as information centrality (the flow of information between any two nodes in a connected network)^66,70,71^. Degree distribution revealed a total of 50 hubs, nodes with ≥ 5 connections within the co-expression network (Fig. 5B; Table S8).

Out of these identified hub genes, co-expression network upregulated hub genes included Aquaporin TIP4-1, Nitrate/peptide (NRT1/ PTR) transporter, Ammonium transporter 1 member, vacuolar calcium ion transporter, CDGSH iron-sulfur cluster binding protein, 3-isopropylmalate dehydratase and nitrite reductase, while downregulated genes included sugar transport protein 13, NAT3 (Nucleobase-ascorbate transporter 3 ion symporter) and zinc transporter genes. GO term analysis of these hub genes revealed their involvement in transport processes. Plants use a wide array of chemicals as weapons for their defense against pathogens. These chemicals or compounds can accumulate at high levels in the infected or attacked tissue^72^. Proteins involved in transport processes are vital for long distance transport of these defense compounds to the other tissues and for plant sustainability under viral attack^73^. The differentially expressed transporters identified here might have a role in transport of secondary metabolites and defense related compounds that further activate other mechanisms involved in plant defense responses under viral attack.

Moreover, we determined that the average degree of turquoise and blue modules was constant. We did not observe any change in the degree for blue and turquoise modules compared to the entire co-expression network or blue module. Intriguingly, we also found that the turquoise module exhibited a significantly heightened average clustering coefficient (Fig. 5C,D). Finally, our information centrality measure in the largest component with 92 nodes determined that the turquoise module displayed significantly increased information centrality compared to the entire network (Fig. 5E; Table S9). These data suggest that nodes in the network may transmit the information via DEGs faster.

### Oxidative stress related genes under CLCuD infection

Light has a major role in photosynthesis and meeting energy demands of the plant under pathogen attack. It provides energy as well as elicits protection to plants against invading microbes^74^. We found GO enrichment of light and radiation based gene expression under a module after co-expression network analysis (Figure S5). Under plant microbe attack, one protective strategy in plants is to produce reactive oxygen species (ROS)^75^. Nicotinamide adenine dinucleotide (reduced form NADH) and cytochrome oxidase (COX) enzymes are involved in synthesis of glutathione S-transferase a major scavenger of ROS. However, another mechanism involved in generation of ROS under biotic stress includes light dependent ROS production from chloroplasts^74^. Therefore, upregulation of COX1, COX3, NADH and genes related to light response in asymptomatic *G. arboreum* plants suggests modulation of gene regulation to combat CLCuD by ROS scavenging.

### Role of primary metabolic pathways in plant immunity

Cellular energy demands have been found to be increased under pathogen attacks in plants. Primary metabolic pathways along with secondary metabolic processes support these increased requirements of the plants. Secondary metabolites including terpenes and phenolics are required by plants for protection against microbial pathogens. However, plant secondary metabolic pathways create a large carbon flux by influencing and reconfiguring the primary metabolism for resistance responses^76,77^. We identified upregulation of genes related to the metabolic pathway specifically involved in steroid and lipid metabolism in asymptomatic plants (Figure S6). These genes include flavonoids, brassinosteroid, sphingolipids and terpenoids, suggesting a role in the defense response to CLCuD. Carbohydrates or sugars act as a fuel to boost energy levels in plants during plant microbe interaction. These are studied as potent players in coordinated plant metabolism with development as well as dealing with different stress responses^78^. The concept of “sweet immunity” has been established based on their importance in plant immunity. In the present dataset, several genes involved in carbohydrate or saccharide metabolism were found to be highly expressed including phosphoenolpyruvate carboxykinase, fructose-1 6-bisphosphatase and phosphoglucomutase (Figure S6). We detected several genes related to cation or meta ion channel activity by WGCNA (Figure S6). Ca2 + signaling related genes, including calmodulins (CaCML) and cyclic nucleotide-gated ion channels (CNGs), were elevated with significantly higher expression levels during CLCuV infection. The upregulation of these ion channels is correlated with the previous studies where cytosolic Ca2 + levels have been observed to be triggered under pathogen infection. Furthermore, Ca2 + acts as second messenger that induces defense related signaling pathways^79,80^. In the blue module, we identified upregulated genes in asymptomatic plants related to metabolic pathways specifically involved in steroid and lipid metabolism (Figure S5). These genes encode flavonoids, brassinosteroids, sphingolipids and terpenoids, known to play a role in defense response.

In this study, we extended the fundamental understanding of *G. arboreum’s* response to CLCuD infection. *G. arboreum’s* natural resistance to CLCuD may involve a complicated gene network, which starts with a basal response and production of general pathogen-associated molecular pattern molecules, followed by activation of CLCuD defense response signaling cascades leading to the transport of defense compounds to other plant tissues and all these interconnected layers of responses ultimately cause immunity to CLCuD in *G. arboreum*. The transcriptomic data provided here is a valuable resource to further characterize candidate genes that are responsive to CLCuD infection and involved in the resistance gene network; these genes can be used in future breeding programs using new breeding techniques like CRISPR to engineer resistance against geminiviruses^81–83^. Consequently, this study will help the scientific community to better understand the mechanisms of *G. arboreum* resistance against CLCuD.

## Materials and Methods

### Plant growth and virus inoculation by grafting

Plants of *G. arboreum*, variety Ravi, were grown in an insect-free glasshouse. *G. hirsutum*, variety CIM 496, plants were maintained in a separate glasshouse and were inoculated with CLCuD by allowing viruliferous whiteflies to feed on these plants. Severely CLCuD infected CIM496 branches were then used as scions to graft on Ravi plants. *G. arboreum* plants were grafted using a “bottle shoot” grafting method^30,32^. In this technique, a graft-scion is placed in a tube filled with distilled water to support plant growth at the higher temperatures maintained for this experiment (Fig. 1). The water in the tube was refreshed daily till the graft union between root stock and scion was established. The tube of water was removed at day 9 after grafting. The temperature of the glasshouse was maintained between 38–45 °C for day time and 25–30 °C for night time. The leaf tissue was collected from CLCuD symptomatic and asymptomatic plants at day 25 post graft infestation. Three biological replicates were processed independently.

### RNA extraction

Total RNA was extracted from CLCuD infested symptomatic and asymptomatic leaves using TRIzol reagent according to the manufacturer’s instructions (Invitrogen, Carlsbad, CA, USA). Three biological replicates from each sample were used for this experiment. The quality and quantity of RNA were assessed by electrophoresis on 1% agarose gels and by a NanoDrop 1000 spectrophotometer (Thermo Fisher Scientific, USA). The integrity of RNA samples was examined using Bio-analyzer 2100 equipment (Agilent Technologies, Germany).

### Library construction and RNA sequencing

The extracted total RNA samples with a concentration of 10 µg were used for cDNA synthesis and strand specific RNA-Seq libraries were constructed as described earlier^84^. Poly (A) mRNA was isolated using oligo-dT beads (Qiagen, Hilden, Germany). The mRNA was broken into short fragments (~300 nt). First-strand cDNA was synthesized using random hexamer-primed reverse transcription. Second-strand cDNA was generated using RNase H and DNA polymerase I. The cDNA fragments were purified and washed for end repair and ligated to sequencing adapters. The cDNA fragments of suitable size were purified and enriched by PCR to obtain the final cDNA library. The integrity of each cDNA library was examined using Bio-analyzer 2100 equipment (Agilent Technologies, Germany). The cDNA libraries were then sequenced using single-end mode of HiSeq™ 2500 equipment (Illumina, San Diego, CA, USA).

### RNA-Seq data analysis

Clean reads were selected after preprocessing with Trimmomatic^85^ to remove low-quality sequences (*i.e*. reads containing adaptor sequences, and reads with more than 5% unknown bases). After preprocessing the RNA-Seq data, the quality of reads was checked by FastQC^86^. The cleaned reads were then mapped to the reference *G. arboreum* genome^87^ using HISAT2-build and HISAT2 aligner^88^. Default HISAT2 parameters, which allow up to two mismatches and report up to 20 alignments for reads mapping at multiple positions, were used. The sequence alignment/map files generated by HISAT2 were used as the input to the software Cufflinks^34^ which assembles the alignments in the sequence alignment/map file into transfrags. Cufflinks does this assembly independently of the existing gene annotations and constructs a minimum set of transcripts that best describes the RNA-Seq reads. The unit of measurement used by Cufflinks to estimate transcript abundance is FPKM (Fragments Per Kilobase of transcript per Million mapped reads). The Cufflinks statistical model probabilistically assigns reads to the assembled isoforms. Cuffdiff was used to find differentially expressed genes (DEGs). The read coverage of one gene was used to calculate the gene expression level, which was measured with the fragment per kilobase of exon model per million mapped reads (FPKM) method. A q value cutoff of 0.05 was used to determine whether a gene had differential expression between samples.

### Quantitative real time PCR

To verify the differential expression detected by the Illumina RNA-Seq data, quantitative real-time RT-PCR (qRT-PCR) was performed on a new set of symptomatic and asymptomatic samples. A set of 17 genes was chosen, including 10 upregulated and 7 downregulated genes (Table S4). Primers for qPCR were designed with the program Primer3 (http://bioinfo.ut.ee/primer3-0.4.0/primer3) with default settings. All primer sequences are provided in Table S3. qRT-PCR was performed using a QUANTSTUDIO 6 flex qRT-PCR instrument and the light cycler fast start DNA Master SYBR Green I kit (Roche, Basel, Switzerland). Reactions were performed in triplicate, and contained 100 ng of cDNA, 0.5 μ L of each primer (10 μ M/μ L), and 10 μ L SYBR Green Master Mix in a final volume of 20 μ L. The amplification reactions were performed under the following conditions: 95 °C for 5 min, followed by 40 cycles of 95 °C for 15 s, 55 °C for 20 s, and 72 °C for 30 s. Melting curve analysis, performed by increasing the temperature from 55 to 95 °C (0.5 °C per 10 s), and gel electrophoresis of the final product confirmed the presence of single amplicons. Relative fold differences for each sample in each experiment were calculated using the ΔΔ Ct method and the amplification of 18 S rRNA was used as an internal control to normalize all data^89^. To corroborate the expression levels measured by RNA-Seq, the ratio of expression as measured by qRT-PCR was compared to the ratio of expression levels between samples using RNA-Seq.

### Construction of co-expression network and network analyses

To construct a co-expression network, we processed transcripts with FPKM count ≥10 and removed all the outliers, which yielded a total number of 468 DEGs. We implemented the R-based Weighted Gene Co-expression Network Analysis (WGCNA)^67,68^ package to construct a co-expression network. The dendrogram was constructed using the cutreeDynamicTree algorithm^68^, with a threshold of minimum module size of 70 genes. A weighted correlation threshold of ≥0.85 was set that resulted in the identification of two modules representing the entire network. Python-based NetworkX^90^ and Cytoscape v. 3.5.1 Plugins^91^ were utilized for network analyses including information centrality, degree and cluster coefficients. The weighted co-expression network was visualized using “Group Attributes Layout”, a feature of Cytoscape v. 3.5.1^92^. Box plot was used to display the distribution of nodes within each module for information centrality, degree and cluster coefficients. Students *t*-test describes statistical significance.

### Gene ontology and functional annotation of DEGs

To determine the main biological functions and pathways of the DEGs, all DEGs were mapped to terms in the Kyoto Encyclopedia of Genes and Genomes (KEGG: www.kegg.jp/kegg/kegg1.html) databases^93^ using AgriGO and Kobas 3.0 tools respectively. Functional analysis of DEGs was done by searching the gene IDs of DEGs in the Swissprot and TrEMBL blast alignment hits available on Cottongen database (https://www.cottongen.org/). Furthermore, the GO term analysis of the identified nodes and hub genes in WGCNA was performed to determine their possible roles in plant defense to CLCuD.

### Data availability

RNA-Seq data from this study have been deposited at the NCBI under the BioProject accession No. PRJNA380937 and BioSample Nos. SAMN06628122, SAMN06628123, SAMN06628124, SAMN06628125, SAMN06628126 and SAMN06628127.

## Electronic supplementary material

Supplementary information

Table S2

Table S8

Table S9
