# Supplementary material for: Transcriptomics reveals multiple resistance mechanisms against cotton leaf curl disease in a naturally immune cotton species, Gossypium arboreum
Source: Sci Rep. 2017 Nov 21;7:15880. doi: 10.1038/s41598-017-15963-9 (PMC5698292; doi:10.1038/s41598-017-15963-9)
Supplement: Supplementary file 1 — Supplementary information [file 41598_2017_15963_MOESM1_ESM.pdf]

Supplementary information

Transcriptomics reveals multiple resistance mechanisms against cotton leaf curl disease in a naturally immune cotton species, *Gossypium arboreum*

Rubab Zahra Naqvi[1,2,3†], Syed Shan-e-Ali Zaidi[1,2,3,8†] (ORCID 0000-0002-2976-4624), Khalid Pervaiz Akhtar[4], Susan Strickler[3], Melkamu Woldemariam[3], Bharat Mishra[5], M. Shahid Mukhtar[5], Brian E. Scheffler[6], Jodi A. Scheffler[7], Georg Jander[3], Lukas A. Mueller[3], Muhammad Asif[1] and Shahid Mansoor[1*]

[1]Agricultural Biotechnology Division, National Institute for Biotechnology and Genetic Engineering (NIBGE), Jhang Road, Faisalabad, Punjab, Pakistan
[2]Pakistan Institute of Engineering & Applied Sciences (PIEAS), Nilore, Islamabad, Pakistan
[3]Boyce Thompson Institute, 533 Tower Road, Cornell University, Ithaca, NY, USA
[4] Nuclear Institute for Agriculture & Biology (NIAB), Jhang Road, Faisalabad, Punjab, Pakistan
[5]Department of Biology, University of Alabama at Birmingham, Birmingham, AL, USA
[6]Genomics and Bioinformatics Research Unit Stoneville, MS, USA
[7]Crop Genetics Research Unit, United States Department of Agriculture-Agricultural Research Service (USDA-ARS), Stoneville, MS, USA
[8]Present address: AgroBioChem Department, Gembloux Agro-Bio Tech, University of Liège, 5030 Gembloux, Belgium

[†]These authors contributed equally to this work.

***Correspondence:**
Shahid Mansoor
shahidmansoor7@gmail.com

Supplementary Discussion

Validation of the transcriptome data by qRT-PCR

Based on log_2fold-change, p_values and q_values of RNA-Seq data, we selected a set of 17 representative genes. We designed primers and performed qPCR on cDNA of independent biological replicates of *G. arboreum*. Our qPCR data strongly correlated with the RNA-Seq expression data (**Figure 4**). The detailed description of these genes and their possible involvement in plant defense response is elaborated below.

Resistance gene analog

In our RNA-Seq and qPCR data, we identified a putative disease resistance protein RGA3 (Cotton_A_03097_BGI-A2_v1.0). RGAs are the candidate R-genes that have conserved motifs and domains and these have specific roles in plant resistance to diverse pathogens (Sekhwal et al., 2015). As a part of their effective innate immune system, plant genomes carry R-genes that function in pathogen recognition and defense response signal transduction. At present, several disease resistance *R* genes have been cloned from higher plants (Zipfel, 2008). Most dominant of these R-genes in monocots and dicots belong to NBS-LRR immune receptors. This class of R genes that have well conserved roles in higher plants that are to guard the plant cells against different bacterial, fungal and viral diseases (Song et al., 2003; Van Der Vossen et al., 2003; Chen et al., 2015). R gene-triggered resistance is associated with a rapid defense response known as hypersensitive response (HR) that can bring a localized cell death at the site of infection, relaying a series of downstream defense pathways (Thomma et al., 2011). R-genes have been found to be induced in response to several pathogens and diseases in plants, including tobacco mosaic virus rice blast, Arabidopsis downy mildew, tomato leaf mould and Verticillium wilt-resistance in cotton (Chen et al., 2015). The decreased levels of RGA in *G. arboreum* contrasts with the previous studies, and it might have a role in symptom development of CLCuD on cotton.

Phytosulfokines

Phytosulfokines 3 was among the well downregulated and qPCR validated genes in our dataset (Cotton_A_25246_BGI-A2_v1.0). In *Arabidopsis thaliana* phytosulfokines (PSKs) have been studied to be perceived by leucine-rich repeat receptor (LRR) kinases and involved in wounding, pathogens and elicitor responses (Loivamäki et al., 2010). PSKs also attenuate the pattern-triggered immunity (PTI) that is triggered by recognition of pathogen elicitors termed as microbe-associated molecular patterns (MAMPs). Even though, immune responses may provide good protection to plants from pathogens but the excessive immune responses may have a negative impact on plant growth and development. Thus, a good balance between positive and negative effects on the immune signaling network is important for plant fitness. PSK genes are therefore, involved in attenuation of PTI signaling in plants, however it stimulates plant growth

and development (Igarashi et al., 2012); (Wang et al., 2015b). Our results suggest that downregulation of PSK in *G. arboreum* has role in increasing the plant immunity to CLCuD.

Boron transporter

Boron is essential element and is required in a certain concentration for the growth and development of plant (Takano et al., 2002). We identified and validated the upregulation of Boron transporter 4 in *G. arboreum* (Cotton_A_28295_BGI-A2_v1.0). In *A. thaliana,* the boron transporter AtBOR1 is localized in the plasma membrane and regulates boron concentration in xylem tissues, to protect shoots from boron toxicity and necrosis (Takano et al., 2005). Boron transporter acts as a host factor to recognize the *Alternanthera mosaic virus* coat protein as an elicitor of systemic necrosis in *Nicotiana benthamiana*. This interaction of host boron transporter with viral coat protein regulates boron transport to induce necrosis at low temperatures, or that the interaction modulates MAP kinases based R gene response (Lim et al., 2014). Based on these studies the upregulation of boron transporter gene in *G. arboreum* depicts the protection of the plant from boron toxicity. Also in *G. arboreum* boron transporter may also be involved in host-viral interaction which further modulates the R-gene mediated response in host to combat virus infection.

Nitrate reductase

We identified and validated the upregulation of nitrate reductase [NADH] in CLCuD infected *G. arboreum* (Cotton_A_23939_BGI-A2_v1.0). Nitrate reductase (NR) has a role in plant pathogen interaction. It is one of the key enzymes producing nitric oxide (NO), that is involved in activating disease resistance in plants, and it is induced by fungal pathogen infection to in potato and tobacco (Yamamoto-Katou et al., 2006; Fagard et al., 2014). NR mutants of *A. thaliana* showed decreased arginine and nitrite levels, impaired nitric oxide synthesis and the hypersensitive response to bacteria pathogen *Pseudomonas syringae* (Modolo et al., 2006). The increased level of NR in *G. arboreum* may thus be associated with the NO mediated disease resistance and ultimately an immune response to CLCuV.

Small heat shock protein

A Small heat shock protein C2 (Cotton_A_29104_BGI-A2_v1.0) was upregulated in symptomatic *G. arboreum* plants. Induction and protein accumulation of small heat shock proteins in plants during several abiotic and biotic stresses shows the role of these proteins in stress tolerance (Sun et al., 2002). A microarray study revealed that defense related genes along with small heat shock genes are coordinately regulated in response to viruses including cucumber mosaic cucumovirus, oil seed rape tobamovirus, turnip vein clearing tobamovirus, potato virus X potexvirus, and turnip mosaic potyvirus viral infection (Whitham et al., 2003; Mandadi and Scholthof, 2013). The gene expression of heat shock proteins in plants is usually upregulated in response to environmental stresses like temperature, salt, light and microbial pathogens (Maimbo et al., 2007). However, genome-wide transcription analysis in *A. thaliana*

was done under the infestation of *E. coli* which lead to a downregulation of heat shock proteins. This absence of heat shock protein induction was correlated with the uptake and subsequent microbial digestion that helps plant in resources re allocation to remain competitive during stress (Paungfoo-Lonhienne et al., 2010). The downregulation of small heat shock protein in *G. arboreum* asymptomatic leaves during CLCuV infestation may indicates its role in viral degradation and helping plant to remain viable during this stress.

***ECERIFERUM1* (*CER1*)**

Aerial organs f land plants are covered by cuticle that is a hydrophobic layer or barrier to protect plants from ultraviolet radiation, desiccation and pathogens. Plant cuticle is mainly consisted of waxes that are long chain alkanes (Eigenbrode et al., 2000). *CER1* controls alkane biosynthesis and is so involved in plant defense responses to several biotic and abiotic stresses. Arabidopsis *ECERIFERUM1* (*CER1*) overexpression is associated with a dramatic increase in the production of the odd-carbon-numbered alkanes, with a substantial accumulation of iso-branched alkanes. Therefore, C*ER1* overexpression in plants showed increased susceptibility to bacterial and fungal pathogens (Bourdenx et al., 2011).The upregulation of asymptomatic *G. arboreum ECERIFERUM1 indicates its involvement in plant pathogen interaction and* resistance of plant to CLCuV.

bHLH transcription factor UNE10

Transcription factor UNE10 belongs to the basic helix-loop-helix (bHLH) proteins superfamily. This family of transcription factors can bind to specific DNA target sites. Several bHLH proteins have been identified and well characterized *Arabidopsis* and rice and have shown important regulatory role in different biological processes including hormone signaling, anther and epidermal cell development, regulation of fruit dehiscence and stress responses (Feller et al., 2011). A recent study on genome-wide analysis of bHLH transcription factor in *Solanum lycopersicum* shows that bHLH transcription factors are involved in the plant defense under infection by tomato yellow leaf curl virus and upregulation of bHLH is associated with the disease resistance (Wang et al., 2015a). *G. arboreum* transcription factor UNE10 was up regulated during CLCuD stress suggest its role in disease resistance.

Potassium channel AKT2

Potassium is an inorganic solute that is abundantly present in plants. Previous studies have shown that altering the potassium status reduces the viral diseases in plants. Potassium channels (AKT) respond to potassium levels in plants as well as other hormonal and environmental stimuli (Wang and Wu, 2010). Thus, alteration in the expression level of potassium channels is associated with viral disease resistance. Significantly enhanced soybean mosaic virus resistance was observed in soybean by the overexpression of GmAKT2 (Zhou et al., 2014). Thus, the upregulation of *G. arboreum* AKT2 gene might have a role in CLCuD tolerance.

Defensin

G. arboreum has showed a higher level of defensing gene expression against CLCuD. Plant defensins are essential components of host defense against pathogen resistance especially bacterial, fungal and insects (Thomma et al., 2002). Defensins are distributed consistently in leaves, flowers, seeds and therefore and is believed that such places are the first contact with a potential pathogens (Lacerda et al., 2014). The integral role of defensins is in the plant innate immune system and mostly plant defensins show an up regulation in their expression under abiotic as well as biotic stresses like injury and pathogen attack (De Beer and Vivier, 2011).

Zinc metallopeptidase EGY3

This protein belongs to matrix metalloproteinases (MMPs) which are a family of zinc-dependent endopeptidases. A few MMPs have been characterized in plants and are involved in plant growth, development and stress responses (Marino and Funk, 2012). Transcript levels increased have been shown to rapidly increase while interaction with pathogens in tobacco and *Medicago truncatula* under the infection of *Pseudomonas syringae and Sinorhizobium meliloti respectively* (Combier et al., 2007; Schiermeyer et al., 2009). The upregulation of this gene in *G. arboreum* is an indication of its contribution in plant defense response to pathogen.

Vignain

Vignain belongs to plant proteases that have been found to have function against pathogens by activation of the defense mechanism. An estimated 3% of the genome in Arabidopsis encodes proteases. Proteases are involved in processing the virulence effectors that are delivered by pathogens. This proteolytic processing further activates the basal defense mechanism (Xia, 2004). It was studied that CDR1 gene in Arabidopsis encodes a protease that generates a mobile endogenous peptide elicitor to activate the basal defense mechanism. Studies have revealed that gene expression level of proteases are induced following pathogen infection in plants. A*rabidopsis* protease has been hypothesized to have a role in activating downstream immune signaling processes upon fungal attack (Figueiredo et al., 2014). The down regulation of vignain protease in *G. arboreum* might have its role in plant virus interaction.

Transcription factor RADIALIS

The RAD gene in plants encodes a single-repeat MYB transcription factors. The MYB gene family is ubiquitous in eukaryotes and are one of the richest groups of transcription factors in plants. Plant MYB proteins are characterized by a highly conserved MYB DNA-binding domain(Stevenson et al., 2006). MYB transcription factors act as master regulators of cellular responses and are involved in different plant development, secondary metabolism, hormone signal transduction, abiotic stress tolerance and disease resistance. These transcription factors have been studied to be involved in mechanisms of disease resistance in several plants through

regulating defense genes(Katiyar et al., 2012). Knockdown of a MYB gene in Arabidopsis enhanced its susceptibility to white cabbage butterfly (*Pieris rapae*)(De Vos et al., 2006). In tobacco, overexpressing a wheat MYB gene showed significantly enhanced resistance to the pathogen *Ralstonia solanacearum,* and transgenic plants were also having increased tolerance to salt and drought stresses (Liu et al., 2011a). According to another study MYB transcription factors platy important role in plant metabolism and photosynthesis (Saibo et al., 2009). Plants reallocate its energy from photosynthesis during responses to pathogen stress (Yuan et al., 2005). Decreased metabolic activity has observed along with downregulation of MYB in rice under brown planthopper infection (Wang et al., 2012). Therefore, the downregulation of *G. arboreum* MYB in our experiment may indicate the activation of other stress responsive genes and reallocation of pant energy to prevent further damage related to loss of water and nutrients.

Bidirectional sugar transporter SWEET17

SWEETs are bidirectional vacuolar fructose transporters in plants predicted to be involved in maintaining sugar homeostasis in plant organs during favorable and under abiotic and biotic stressed conditions. SWEET transporters have been found to be associated with plant pathogen interactions during a pathogen attack in plants (Chandran, 2015). Pathogens mainly infect plant cells to fuel their own growth and reproduction by acquiring plant nutrients, especially sugars that often damages the plant productivity (Chakraborty and Newton, 2011). Studies in rice have revealed that bacterial pathogens like bacterial blight disease causing *Xanthomonas* species mainly co-opt with plant SWEET transporters to access host sugar reservoirs (Antony et al., 2010; Liu et al., 2011b). These bacteria use their type III secretion system to secrete transcription-activator like (TAL) effectors to target the expression of certain *SWEET* genes (Bogdanove, 2014). This targeted induction of SWEETs appears to be required for pathogen growth as lack of induction, in most cases, results in reduced pathogen growth and disease resistance. A Reduced level of *SWEET* gene expression in tobacco and rice have showed enhanced resistance to pathogen, suggesting its role in pathogen growth and plant disease resistance (Antony et al., 2010; Chandran, 2015). The downregulation of *G. arboreum* SWEET transporter suggests its role in sugar homeostasis in unfavorable conditions and possible involvement in reduced pathogen growth and disease resistance.

Chaperone protein DnaJ

DnaJ also known HSP40 or J-domain-containing protein or J-protein is a co-chaperone and component of the HSP70 chaperone machinery. HSP40 has a conserved 70 amino acid J-domain that interacts with nucleotide-binding domain of HSP70 to increase its affinity for substrates or clients (Kampinga and Craig, 2010). DnaJ chaperons are repotted to play an important role in virus-plant interaction and viral pathogenesis particularly in viral movement. It interacts with viral coat or movement protein that as a cascade binds to viral nucleic. This binding of chaperone with viral nucleic acid or virion facilitates the viral movement from cytosol to membrane or plasmodesmata between two cells, which leads to a rapid and severe symptom development in

the plant (Chen et al., 2008; Verchot, 2012). The coat protein of *Potato virus Y* interacts with DnaJ-like protein that is important for cell-to-cell viral movement (Hofius et al., 2007). Similarly, the interaction of movement protein of *Tomato spotted wilt virus* with DnaJ-like protein has been studied in tomato (Soellick et al., 2000). DnaJ has been found to have a role in regulation of plant immunity by interaction with HSP70 for example its silencing in soybean enhanced the susceptibility to *Soybean mosaic virus* (Liu and Whitham, 2013);(Park and Seo, 2015). Hence its upregulation in *G. arboreum* symptomatic plants under CLCuD stress directs its role in helping viral cell to cell movement in plant and making plant susceptible to establish symptoms by graft inoculation of CLCuV.

Aquaporin PIP2

Plant aquaporins also termed as water channel proteins are integral regulator of plant water relations (Afzal et al., 2016; Meng et al., 2016). They have essential role in plant growth and development and involve in plant stress responses against various environmental stresses that disturb plant cell nutrient homeostasis and osmotic balance (Matsumoto et al., 2009). During biotic stresses like pathogen infection, plant produces reactive oxygen species as a player of plant immune system. Aquaporin homologs have been highlighted to have a role in plant defense by transporting H_2O2 during pathogen attack (Tian et al., 2016). In a comparative study of citus susceptible and tolerant plants to proteobacterium, *Candidatus Liberibacter*, under pathogen infestation, six aquaporin genes were found to be differentially expressed and were correlated to disease development (Aritua et al., 2013). The induction of 24 aquporin genes have been reported on expression profiling of soybean under *Pseudomonas syringae* infection (Zou et al., 2005). The role of aquaporins in in response to pathogen infection has also been observed in tobacco and grasses against nematode and herbivores (Opperman et al., 1994; Hartley et al., 2015). Studies have shown the involvement of aquaporins in plant-viral interactions, for example aquaporin genes interact with a cucumber mosaic virus (CMV) replication protein that potentially affects CMV replication in the host plant (Kim et al., 2006). The upregulation of *G. arboreum* aquaporin might reveals its role in plant-virus interaction and disease development.

Protein REVEILLE

REVEILLE is a plant Myb-like transcription factor for the integration of circadian clock and hormone signaling. REVEILLE connects the important networks that provides a synchronization of plant growth with changing rhythms of environment (Rawat et al., 2009). The circadian clock regulation modulates plant growth and development and has often been implicated in response to environmental stresses. In *Arabidopsis,* REVEILLE has been studied to have a role in regulation of necrotrophic fungus and freezing tolerance (Cerrudo et al., 2012; Meissner et al., 2013). The upregulation of *G. arboreum* REVEILLE gene might have a role in plant protection by coordination of plant growth with rhythmic changes in the environment during CLCuV stress.

60S ribosomal protein L39

Ribosomes are highly conserved in nature and required for integral cellular activities including cell growth, development and gene regulation. Ribosome is composed of two subunits, four ribosomal RNAs, and 82 associated ribosomal proteins (Barakat et al., 2001; Rogalski et al., 2008). Ribosomal proteins have been reported to have a role in basal resistance against virulent pathogens both in humans and plants (Zhou et al., 2013; Nagaraj et al., 2016) . Recently, studies have shown the correlation of overexpressed ribosomal protein L3 with antiviral and anti-fungal defense in tobacco. Another study has shown that ribosomal protein L12 and L19 are important in nonhost disease resistance in *N. benthamiana* and *A. thaliana* (Di and Tumer, 2005*)*. A study in *G. arboreum* revealed that upregulation of ribosomal protein L18 mediates resistance to Verticillium wilt by inducing salicylic acid signaling pathway (Gong et al., 2017). So, overexpression of ribosomal protein L39 in our experiment might have a role in inducing pathways involved in *G. arboreum* resistance to CLCuD.

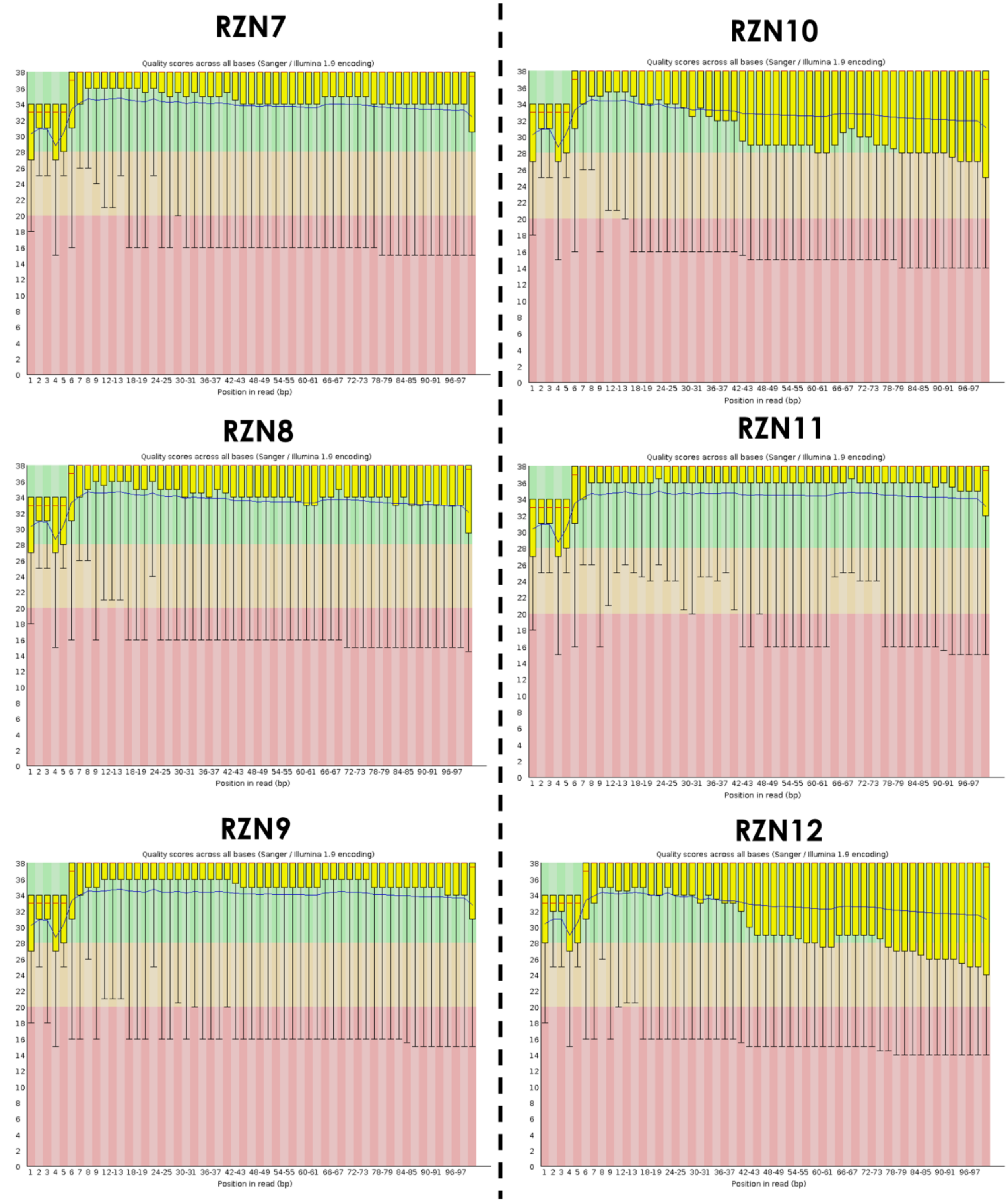

Figure S1: Quality assessment of trimmed FASTQ sequence data

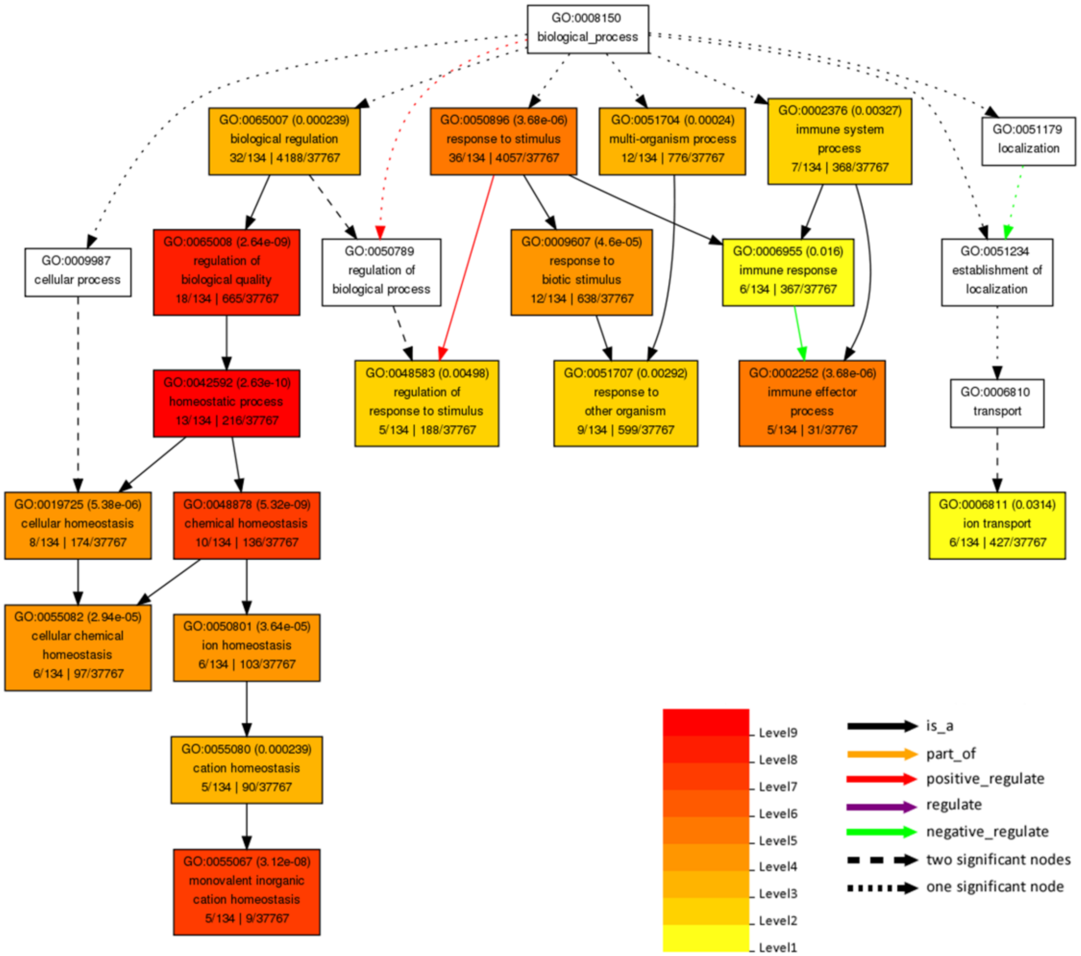

Figure S2: Interactive graph of GO terms associated with cotton leaf curl disease responsive *G. arboreum* differentially expressed genes (top 100). Analysis performed with online tool agriGO (bioinfo.cau.edu.cn/agriGO/) where a key at bottom indicates significance levels of GO terms.

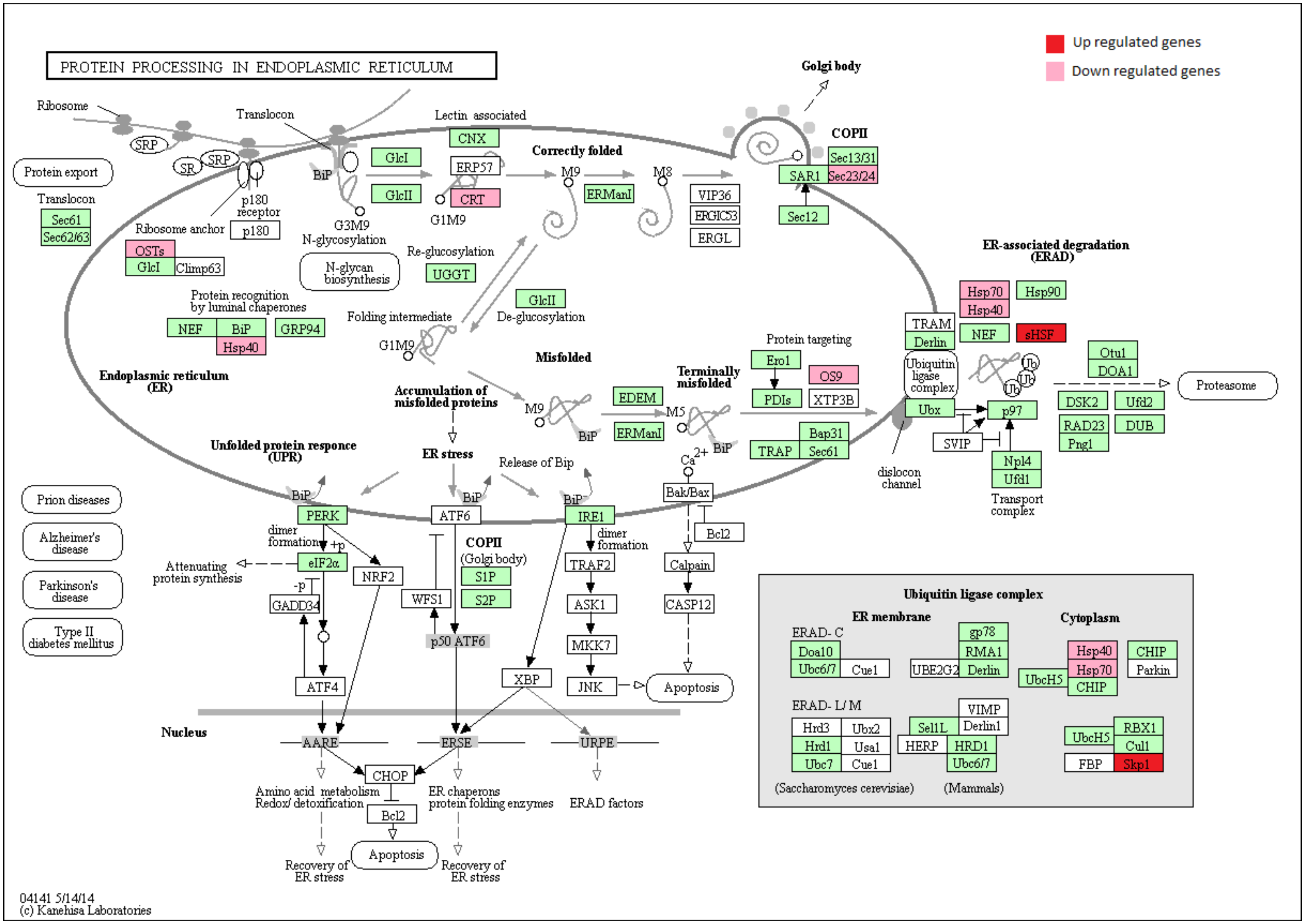

Figure S3: KEGG pathway associated with ubiquitin mediated proteolysis. Pathway analysis was performed using KEGG (Kanehisa et al., 2017: www.kegg.jp/kegg/kegg1.html) and Kobas 3.0.

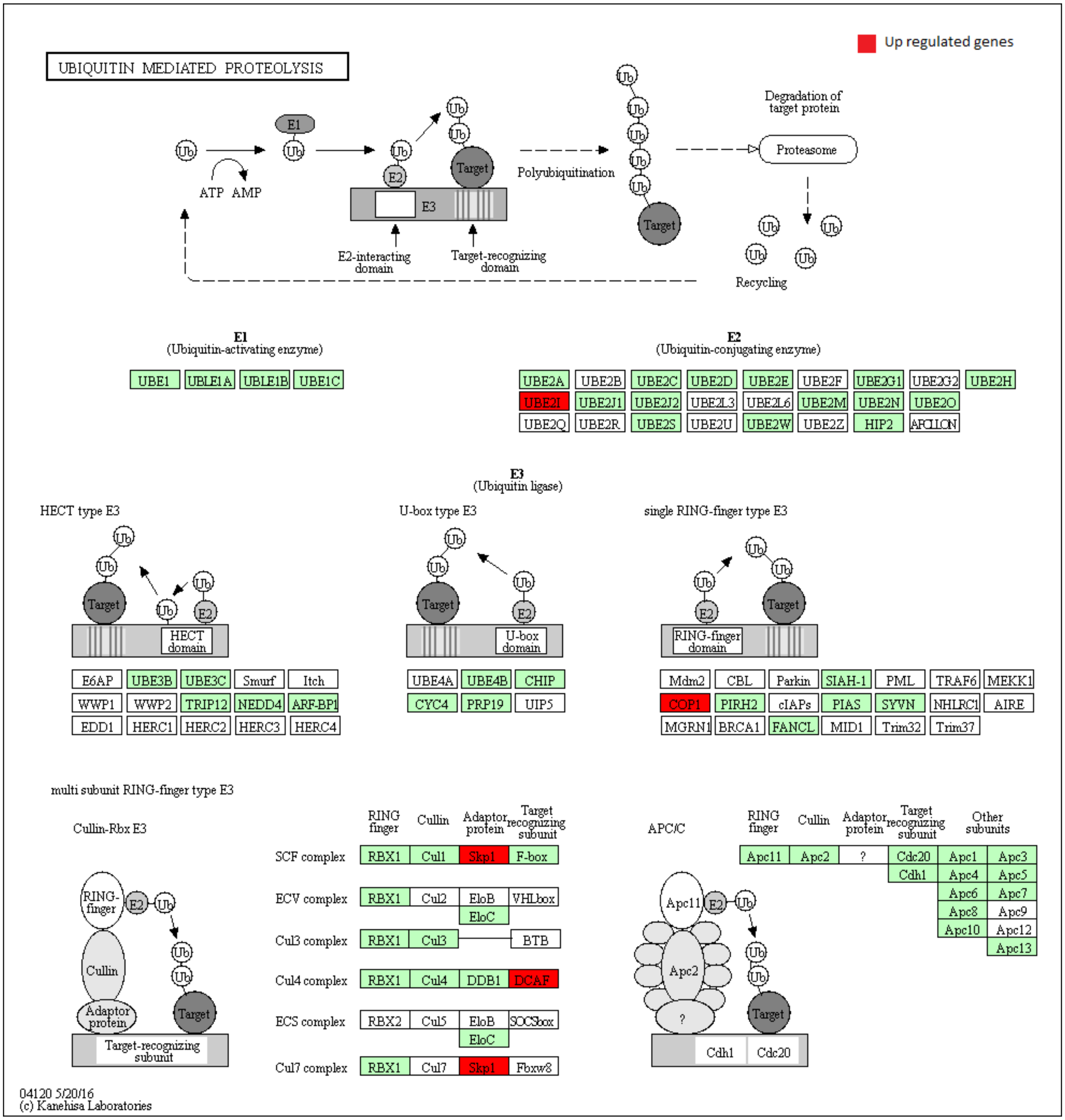

Figure S4: KEGG pathway associated with protein processing in endoplasmic reticulum. Pathway analysis was performed using KEGG (Kanehisa et al., 2017: www.kegg.jp/kegg/kegg1.html) and Kobas 3.0.

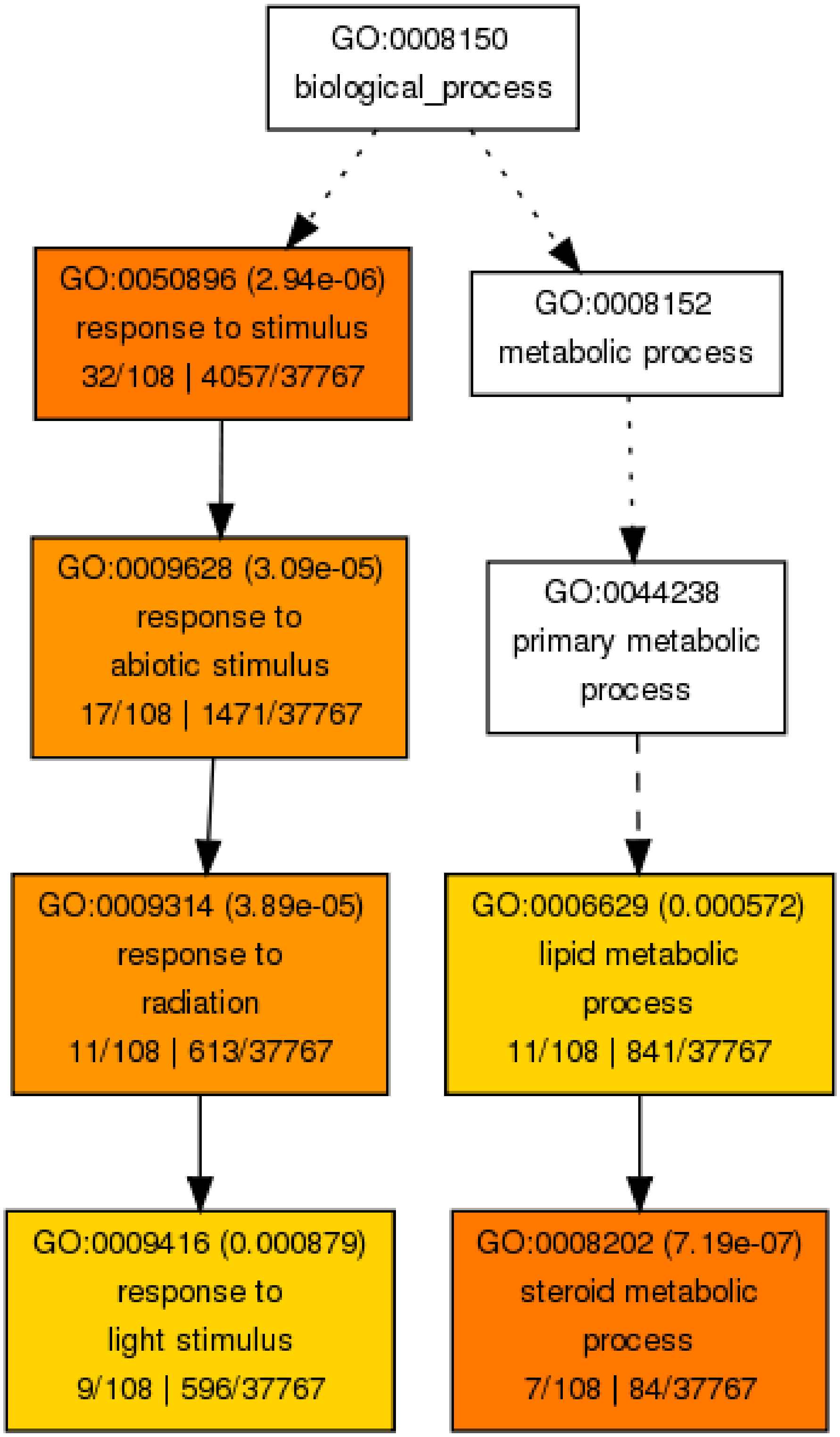

Figure S5: Interactive graph of GO terms associated with blue module identified by co-expression network analysis. GO term analysis performed with online tool agriGO (bioinfo.cau.edu.cn/agriGO/).

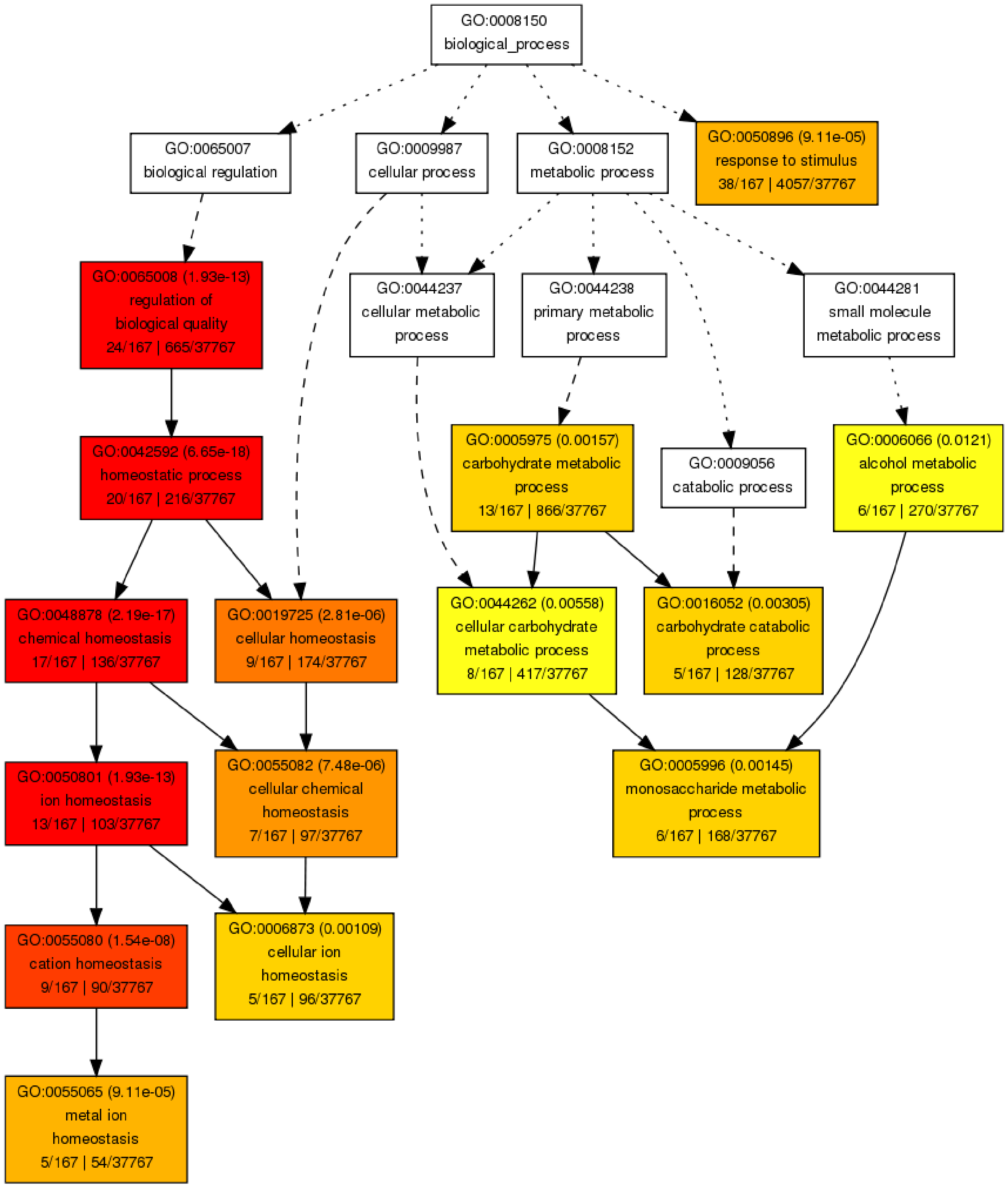

Figure S6: Interactive graph of GO terms associated with turquoise module identified by co-expression network analysis. GO term analysis performed with online tool agriGO (bioinfo.cau.edu.cn/agriGO/).

Table S1: Features of reference Gossypium arboreum genome obtained from https://www.cottongen.org/node/1300251

Analysis Name	Gossypium arboreum (A2) Genome BGI Assembly v2.0 & Annotation v1.0
Method	SOAPdenovo
Source	Illumina HiSeq 2000 reads from various insert size libraries (G. arboreum A genome)
Date performed	2014-05-31
Number of pseudomolecules (chromsomes)	13
Total scaffolds (≥ 2kb)	7,914
Number of Gene models	41,330
Number of miRNA	431
Number of rRNA	10,464
Number of tRNA	2,289
Number of snRNA	7,619

Table S3: qRT-PCR primers for RNA Seq Data validation

**Primer Sequemce**	**Gene_ID**
GGAAAGCGAAACAGCAAGAC	Cotton_A_29104_BGI-A2_v1.0
ATTTGGGAGGTGCAGAACAC	
CACTTTTCATAGCTGCGCTTC	Cotton_A_25246_BGI-A2_v1.0
ACATGAGCAGCAAGGGTTCT	
TTCCCGGTAATCAGTTCTGG	Cotton_A_28295_BGI-A2_v1.0
GAAGAAGGGCAATGGAAACA	
ATCTATCATCTCCGCGTTGG	Cotton_A_34570_BGI-A2_v1.0
GGAATCGGTTCCTTTCAACA	
AGAACCCCATTGCACATAGC	Cotton_A_00757_BGI-A2_v1.0
CTTTGTGCAGCAAGGCATAA	
GTGGTGGAGGGCAGAATATG	Cotton_A_17591_BGI-A2_v1.0
CAGTTCCTAGTACAGAAGCAACG	
GTTTCCGGTTACCCTGGTTT	Cotton_A_23939_BGI-A2_v1.0
CTTCAGACCCTTCGAAGCAC	
GCCCCGTCGAGTTATTTGTA	Cotton_A_00108_BGI-A2_v1.0
GTTGGGATTCCCCGTTCTAT	
AAGAAAAGGGCGGATAGGAA	Cotton_A_00151_BGI-A2_v1.0
CCAATGAAAATCCCACCATC	
AGGAAACCAAGGTTGCAATG	Cotton_A_22786_BGI-A2_v1.0
AGCAATGCTTCTTCGTCGTT	
GAGATTGAGGATTGCGGAAA	Cotton_A_03097_BGI-A2_v1.0
CAACCTTTTGGATGTGAGCA	
AGGTTCATGGACAGCCAAAC	Cotton_A_19720_BGI-A2_v1.0
CAGCCACAAGCAGCTCATAG	
ACGTTTTGGCACATCGTACA	Cotton_A_40086_BGI-A2_v1.0
TGGGAGGTGCAAAGATTAGG	
GGACTGGGTGCTGAGATCAT	Cotton_A_07057_BGI-A2_v1.0
GACAGCAGCTCCAAAACTCC	
ATGAGGCAGAACAGGCCTATCCC	Cotton_A_11914_BGI-A2_v1.0
TTAGAACCCTAGCTTGGTACGGCGCC	
AAATCCGGTTTGTGCTGTTC	Cotton_A_01472_BGI-A2_v1.0
CAGCAGTTTTCGTGCCTACA	
CGGCTACCACATCCAAGGAA	18S-internal control
TGTCACTACCTCCCCGTGTCA	

Table S4: Selected differentially expressed genes for qRT-PCR and their differential gene expression

gene_id	locus	value_1	value_2	log2FC	p_value	q_value
Cotton_A_00108_BGI-A2_v1.0	gnl|BGIA2|CA_chr7:38191739-38194551	117.789	1.52922	-6.26727	0.00135	0.032047
Cotton_A_00151_BGI-A2_v1.0	gnl|BGIA2|CA_chr7:38612815-38616206	2.78665	65.9097	4.56389	0.00075	0.021628
Cotton_A_00757_BGI-A2_v1.0	gnl|BGIA2|CA_chr5:7603021-7605571	5.23619	80.8297	3.9483	5.00E-05	0.004237
Cotton_A_01472_BGI-A2_v1.0	gnl|BGIA2|CA_chr6:119691944-119694178	7.74226	149.129	4.26767	5.00E-05	0.004237
Cotton_A_03097_BGI-A2_v1.0	gnl|BGIA2|CA_chr13:82654606-82654846	64.3806	0	-inf	0.0008	0.022474
Cotton_A_07057_BGI-A2_v1.0	gnl|BGIA2|CA_chr10:88062721-88063889	4.47933	98.8612	4.46405	0.0006	0.018406
Cotton_A_11914_BGI-A2_v1.0	gnl|BGIA2|CA_chr3:22049760-22050000	0	951.119	inf	5.00E-05	0.004237
Cotton_A_17591_BGI-A2_v1.0	gnl|BGIA2|CA_chr12:46317685-46317841	0	191.039	inf	0.0003	0.012406
Cotton_A_19720_BGI-A2_v1.0	gnl|BGIA2|CA_chr13:63435527-63435770	89.0602	0	-inf	0.0001	0.006735
Cotton_A_22786_BGI-A2_v1.0	gnl|BGIA2|CA_chr7:70420912-70422075	239.853	3.24601	-6.20734	0.00105	0.026869
Cotton_A_23939_BGI-A2_v1.0	gnl|BGIA2|CA_chr12:71717597-71721346	5.1819	293.628	5.82437	5.00E-05	0.004237
Cotton_A_25246_BGI-A2_v1.0	gnl|BGIA2|CA_chr8:94985683-94986012	92.5924	0	-inf	0.0008	0.022474
Cotton_A_28295_BGI-A2_v1.0	gnl|BGIA2|CA_chr8:55796825-55800269	7.49608	129.614	4.11194	5.00E-05	0.004237
Cotton_A_29104_BGI-A2_v1.0	gnl|BGIA2|CA_chr11:102055257-102058107	636.537	4.89619	-7.02244	0.0011	0.027568
Cotton_A_34570_BGI-A2_v1.0	gnl|BGIA2|CA_chr8:72452913-72458132	8.78946	138.661	3.97964	5.00E-05	0.004237
Cotton_A_40086_BGI-A2_v1.0	gnl|BGIA2|CA_chr13:134161414-134162613	143.501	0	-inf	5.00E-05	0.004237
Cotton_A_19100_BGI-A2_v1.0	gnl|BGIA2|CA_chr1:82884357-82886990	4.54332	83.986	4.20833	5.00E-05	0.004237

Table S5: Differential gene expression of Ethylene_response-genes in the transcriptomic data of G. arboreum under CLCuD infection

gene_id	locus	value_1	value_2	log2FC	p_value	q_value
Cotton_A_00151_BGI-A2_v1.0	gnl|BGIA2|CA_chr7:38612815-38616206	2.78665	65.9097	4.56389	0.00075	0.021628
Cotton_A_01965_BGI-A2_v1.0	gnl|BGIA2|CA_chr6:29174682-29175480	317.831	56.8102	-2.48404	5.00E-05	0.004237
Cotton_A_04648_BGI-A2_v1.0	gnl|BGIA2|CA_chr10:19937964-19939419	55.7139	322.799	2.53453	5.00E-05	0.004237
Cotton_A_11306_BGI-A2_v1.0	gnl|BGIA2|CA_chr10:19167132-19167714	17.3996	0	_inf	5.00E-05	0.004237
Cotton_A_18053_BGI-A2_v1.0	gnl|BGIA2|CA_chr11:33845449-33846740	15.5048	201.836	3.70239	0.0004	0.01451
Cotton_A_22458_BGI-A2_v1.0	gnl|BGIA2|CA_chr5:25024119-25024779	155.197	31.3229	-2.30881	5.00E-05	0.004237
Cotton_A_38436_BGI-A2_v1.0	gnl|BGIA2|CA_chr8:11447924-11448518	5.7767	0	_inf	0.0008	0.022474

Table S6: Differential gene expression of R genes in the transcriptomic data of G. arboreum under CLCuD infection

gene_id	locus	value_1	value_2	log2FC	p_value	q_value
Cotton_A_00075_BGI-A2_v1.0	gnl|BGIA2|CA_chr7:37858904-37861479	12.0667	2.29477	-2.39461	0.00235	0.046765
Cotton_A_01444_BGI-A2_v1.0	gnl|BGIA2|CA_chr6:119940486-119941236	0	8.21728	inf	5.00E-05	0.004237
Cotton_A_03097_BGI-A2_v1.0	gnl|BGIA2|CA_chr13:82654606-82654846	64.3806	0	_inf	0.0008	0.022474
Cotton_A_07954_BGI-A2_v1.0	gnl|BGIA2|CA_chr6:50014751-50017049	73.5349	24.133	-1.60742	0.0019	0.041007
Cotton_A_13407_BGI-A2_v1.0	gnl|BGIA2|CA_chr6:99993742-99996355	6.92684	25.4078	1.875	0.0022	0.044639
Cotton_A_19774_BGI-A2_v1.0	gnl|BGIA2|CA_chr11:35453737-35456746	16.0415	2.81418	-2.51102	0.00025	0.011261
Cotton_A_21491_BGI-A2_v1.0	gnl|BGIA2|CA_chr4:9410699-9418467	0	4.41402	inf	5.00E-05	0.004237
Cotton_A_27521_BGI-A2_v1.0	gnl|BGIA2|CA_chr8:73311906-73312434	7.61972	0	_inf	0.0004	0.01451
Cotton_A_30374_BGI-A2_v1.0	gnl|BGIA2|CA_chr2:16168065-16168743	0	5.81196	inf	0.0001	0.006735
Cotton_A_33351_BGI-A2_v1.0	gnl|BGIA2|CA_chr4:79293587-79294172	0	8.4326	inf	0.0004	0.01451
Cotton_A_34256_BGI-A2_v1.0	gnl|BGIA2|CA_chr4:120630533-120630977	0	11.9953	inf	0.00085	0.023527

Table S7: Differential gene expression of kinase genes in the transcriptomic data of G. arboreum under CLCuD infection

gene_id	locus	value_1	value_2	log2FC	p_value	q_value
Cotton_A_00489_BGI-A2_v1.0	gnl|BGIA2|CA_chr2:68657365-68662624	23.4456	7.33695	-1.67606	0.00205	0.042988
Cotton_A_01042_BGI-A2_v1.0	gnl|BGIA2|CA_chr13:75050666-75055909	63.0786	17.6709	-1.83577	0.0001	0.006735
Cotton_A_01175_BGI-A2_v1.0	gnl|BGIA2|CA_chr4:33406606-33407959	4.40997	24.712	2.48637	0.0022	0.044639
Cotton_A_01427_BGI-A2_v1.0	gnl|BGIA2|CA_chr6:120105721-120107697	157.439	40.4777	-1.95959	0.00045	0.015611
Cotton_A_02476_BGI-A2_v1.0	gnl|BGIA2|CA_chr11:21025139-21026831	54.1352	12.1407	-2.15672	0.00055	0.017388
Cotton_A_06203_BGI-A2_v1.0	gnl|BGIA2|CA_chr6:68944361-68947654	46.7306	12.9512	-1.85128	0.0014	0.032969
Cotton_A_06973_BGI-A2_v1.0	gnl|BGIA2|CA_chr10:88748841-88751202	136.31	39.1452	-1.79999	0.00035	0.013577
Cotton_A_07353_BGI-A2_v1.0	gnl|BGIA2|CA_chr11:7510593-7516935	27.431	181.735	2.72796	5.00E-05	0.004237
Cotton_A_11474_BGI-A2_v1.0	gnl|BGIA2|CA_chr10:69456460-69458525	57.335	12.1962	-2.23298	0.00045	0.015611
Cotton_A_14230_BGI-A2_v1.0	gnl|BGIA2|CA_chr7:18009881-18019817	55.1628	5.48061	-3.33129	5.00E-05	0.004237
Cotton_A_14231_BGI-A2_v1.0	gnl|BGIA2|CA_chr7:18033096-18040726	23.2418	1.8219	-3.6732	5.00E-05	0.004237
Cotton_A_14232_BGI-A2_v1.0	gnl|BGIA2|CA_chr7:18046613-18054222	9.47296	1.60726	-2.55921	0.00205	0.042988
Cotton_A_15229_BGI-A2_v1.0	gnl|BGIA2|CA_chr1:128036614-128037463	71.8095	10.3863	-2.78949	0.001	0.026106
Cotton_A_15642_BGI-A2_v1.0	gnl|BGIA2|CA_chr4:137204947-137209185	41.2791	249.543	2.5958	0.0001	0.006735
Cotton_A_16517_BGI-A2_v1.0	gnl|BGIA2|CA_chr11:16173771-16179808	41.6616	115.756	1.4743	0.0019	0.041007
Cotton_A_16741_BGI-A2_v1.0	gnl|BGIA2|CA_chr2:60824477-60827881	18.0563	5.27256	-1.77593	0.0017	0.037999
Cotton_A_17344_BGI-A2_v1.0	gnl|BGIA2|CA_chr9:31296716-31297799	133.281	27.5267	-2.27556	5.00E-05	0.004237
Cotton_A_17498_BGI-A2_v1.0	gnl|BGIA2|CA_chr9:12130179-12142499	41.4669	9.68616	-2.09796	5.00E-05	0.004237
Cotton_A_18724_BGI-A2_v1.0	gnl|BGIA2|CA_chr1:27426797-27429215	21.6872	2.83861	-2.93359	0.0006	0.018406
Cotton_A_19047_BGI-A2_v1.0	gnl|BGIA2|CA_chr12:49705252-49707619	39.5819	4.10637	-3.2689	5.00E-05	0.004237
Cotton_A_19049_BGI-A2_v1.0	gnl|BGIA2|CA_chr12:49669667-49672284	72.8806	26.1084	-1.48102	0.0017	0.037999
Cotton_A_20472_BGI-A2_v1.0	gnl|BGIA2|CA_chr1:59147041-59150376	20.1794	4.4171	-2.19172	0.0009	0.024389
Cotton_A_20984_BGI-A2_v1.0	gnl|BGIA2|CA_chr8:63913024-63913447	11.3593	0	_inf	0.002	0.042282
Cotton_A_21151_BGI-A2_v1.0	gnl|BGIA2|CA_chr9:41272351-41275298	51.2317	15.2314	-1.74998	0.0023	0.04608
Cotton_A_21201_BGI-A2_v1.0	gnl|BGIA2|CA_chr3:59714615-59715828	209.042	59.8822	-1.80359	0.00215	0.044057
Cotton_A_22249_BGI-A2_v1.0	gnl|BGIA2|CA_chr8:26498011-26501174	34.6935	10.7718	-1.68741	0.00135	0.032047
Cotton_A_22570_BGI-A2_v1.0	gnl|BGIA2|CA_chr10:12316909-12320944	9.38004	92.9193	3.30831	5.00E-05	0.004237

Cotton_A_23060_BGI-A2_v1.0	gnl|BGIA2|CA_chr9:77857506-77869160	38.1568	5.09921	-2.90359	5.00E-05	0.004237
Cotton_A_25556_BGI-A2_v1.0	gnl|BGIA2|CA_chr12:22027282-22029646	34.365	8.41093	-2.0306	0.0003	0.012406
Cotton_A_25849_BGI-A2_v1.0	gnl|BGIA2|CA_chr13:119412083-119415010	23.579	109.635	2.21713	0.0002	0.009799
Cotton_A_29627_BGI-A2_v1.0	gnl|BGIA2|CA_chr9:43969287-43974380	17.4313	1.31218	-3.73164	0.00235	0.046765
Cotton_A_29772_BGI-A2_v1.0	gnl|BGIA2|CA_chr13:92047203-92047942	0	10.7962	inf	0.00025	0.011261
Cotton_A_29908_BGI-A2_v1.0	gnl|BGIA2|CA_chr2:86072084-86075976	23.0975	80.2823	1.79734	0.00055	0.017388
Cotton_A_30881_BGI-A2_v1.0	gnl|BGIA2|CA_chr8:50885811-50888353	42.0303	6.59963	-2.67097	0.002	0.042282
Cotton_A_30882_BGI-A2_v1.0	gnl|BGIA2|CA_chr8:50895399-50897963	44.138	10.6881	-2.04602	0.00225	0.045342
Cotton_A_30892_BGI-A2_v1.0	gnl|BGIA2|CA_chr8:51192936-51193290	0	28.8302	inf	0.0003	0.012406
Cotton_A_32553_BGI-A2_v1.0	gnl|BGIA2|CA_chr4:42548129-42550968	53.0137	7.57963	-2.80617	5.00E-05	0.004237
Cotton_A_32557_BGI-A2_v1.0	gnl|BGIA2|CA_chr4:42570411-42571788	95.9118	16.5713	-2.53302	5.00E-05	0.004237
Cotton_A_33914_BGI-A2_v1.0	gnl|BGIA2|CA_chr1:11321262-11328543	20.8969	98.5848	2.23807	0.0003	0.012406
Cotton_A_34190_BGI-A2_v1.0	gnl|BGIA2|CA_chr1:49831463-49833962	10.0313	0	_inf	5.00E-05	0.004237
Cotton_A_34636_BGI-A2_v1.0	gnl|BGIA2|CA_chr8:7839155-7841843	57.1537	314.214	2.45883	0.00105	0.026869
Cotton_A_34917_BGI-A2_v1.0	gnl|BGIA2|CA_chr7:94571479-94573667	0	4.6606	inf	5.00E-05	0.004237
Cotton_A_36679_BGI-A2_v1.0	gnl|BGIA2|CA_chr11:99794309-99795041	6.29186	0	_inf	0.0002	0.009799
Cotton_A_37182_BGI-A2_v1.0	gnl|BGIA2|CA_chr1:77639537-77642261	37.8343	8.55246	-2.14528	0.0003	0.012406
Cotton_A_37667_BGI-A2_v1.0	gnl|BGIA2|CA_chr8:104888327-104890618	72.572	9.1965	-2.98026	0.0001	0.006735
Cotton_A_37763_BGI-A2_v1.0	gnl|BGIA2|CA_chr2:30869154-30881875	74.5785	7.94282	-3.23104	5.00E-05	0.004237

Referrences

Afzal, Z., Howton, T., Sun, Y., and Mukhtar, M.S. (2016). The roles of aquaporins in plant stress responses. *Journal of Developmental Biology* 4(1)**,** 9.

Antony, G., Zhou, J., Huang, S., Li, T., Liu, B., White, F., et al. (2010). Rice xa13 recessive resistance to bacterial blight is defeated by induction of the disease susceptibility gene Os-11N3. *The Plant Cell* 22(11)**,** 3864-3876.

Aritua, V., Achor, D., Gmitter, F.G., Albrigo, G., and Wang, N. (2013). Transcriptional and microscopic analyses of citrus stem and root responses to Candidatus Liberibacter asiaticus infection. *PloS one* 8(9)**,** e73742.

Barakat, A., Szick-Miranda, K., Chang, F., Guyot, R., Blanc, G., Cooke, R., et al. (2001). The organization of cytoplasmic ribosomal protein genes in the Arabidopsis genome. *Plant Physiology* 127(2)**,** 398-415.

Bogdanove, A.J. (2014). Principles and applications of TAL effectors for plant physiology and metabolism. *Current opinion in plant biology* 19**,** 99-104.

Bourdenx, B., Bernard, A., Domergue, F., Pascal, S., Léger, A., Roby, D., et al. (2011). Overexpression of Arabidopsis ECERIFERUM1 promotes wax very-long-chain alkane biosynthesis and influences plant response to biotic and abiotic stresses. *Plant physiology* 156(1)**,** 29-45.

Cerrudo, I., Keller, M.M., Cargnel, M.D., Demkura, P.V., de Wit, M., Patitucci, M.S., et al. (2012). Low red/far-red ratios reduce Arabidopsis resistance to Botrytis cinerea and jasmonate responses via a COI1-JAZ10-dependent, salicylic acid-independent mechanism. *Plant Physiology* 158(4)**,** 2042-2052.

Chakraborty, S., and Newton, A.C. (2011). Climate change, plant diseases and food security: an overview. *Plant Pathology* 60(1)**,** 2-14.

Chandran, D. (2015). Co-option of developmentally regulated plant SWEET transporters for pathogen nutrition and abiotic stress tolerance. *IUBMB life* 67(7)**,** 461-471.

Chen, J.-Y., Huang, J.-Q., Li, N.-Y., Ma, X.-F., Wang, J.-L., Liu, C., et al. (2015). Genome-wide analysis of the gene families of resistance gene analogues in cotton and their response to Verticillium wilt. *BMC plant biology* 15(1)**,** 148.

Chen, Z., Zhou, T., Wu, X., Hong, Y., Fan, Z., and Li, H. (2008). Influence of cytoplasmic heat shock protein 70 on viral infection of Nicotiana benthamiana. *Molecular plant pathology* 9(6)**,** 809-817.

Combier, J.-P., Vernié, T., de Billy, F., El Yahyaoui, F., Mathis, R., and Gamas, P. (2007). The MtMMPL1 early nodulin is a novel member of the matrix metalloendoproteinase family with a role in Medicago truncatula infection by Sinorhizobium meliloti. *Plant physiology* 144(2)**,** 703-716.

De Beer, A., and Vivier, M.A. (2011). Four plant defensins from an indigenous South African Brassicaceae species display divergent activities against two test pathogens despite high sequence similarity in the encoding genes. *BMC research notes* 4(1)**,** 459.

De Vos, M., Denekamp, M., Dicke, M., Vuylsteke, M., Van Loon, L., Smeekens, S.C., et al. (2006). The Arabidopsis thaliana transcription factor AtMYB102 functions in defense against the insect herbivore Pieris rapae. *Plant signaling & behavior* 1(6)**,** 305-311.

Di, R., and Tumer, N.E. (2005). Expression of a truncated form of ribosomal protein L3 confers resistance to pokeweed antiviral protein and the Fusarium mycotoxin deoxynivalenol. *Molecular plant-microbe interactions* 18(8)**,** 762-770.
Eigenbrode, S.D., Rayor, L., Chow, J., and Latty, P. (2000). Effects of wax bloom variation in Brassica oleracea on foraging by a vespid wasp. *Entomologia Experimentalis et Applicata* 97(2)**,** 161-166.
Fagard, M., Launay, A., Clément, G., Courtial, J., Dellagi, A., Farjad, M., et al. (2014). Nitrogen metabolism meets phytopathology. *Journal of experimental botany* 65(19)**,** 5643-5656.
Feller, A., Machemer, K., Braun, E.L., and Grotewold, E. (2011). Evolutionary and comparative analysis of MYB and bHLH plant transcription factors. *The Plant Journal* 66(1)**,** 94-116.
Figueiredo, A., Monteiro, F., and Sebastiana, M. (2014). Subtilisin-like proteases in plant–pathogen recognition and immune priming: a perspective. *Frontiers in plant science* 5**,** 739.
Gong, Q., Yang, Z., Wang, X., Butt, H.I., Chen, E., He, S., et al. (2017). Salicylic acid-related cotton (Gossypium arboreum) ribosomal protein GaRPL18 contributes to resistance to Verticillium dahliae. *BMC Plant Biology* 17(1)**,** 59.
Hartley, S.E., Fitt, R.N., McLarnon, E.L., and Wade, R.N. (2015). Defending the leaf surface: intra-and inter-specific differences in silicon deposition in grasses in response to damage and silicon supply. *Frontiers in plant science* 6.
Hofius, D., Maier, A.T., Dietrich, C., Jungkunz, I., Börnke, F., Maiss, E., et al. (2007). Capsid protein-mediated recruitment of host DnaJ-like proteins is required for Potato virus Y infection in tobacco plants. *Journal of virology* 81(21)**,** 11870-11880.
Igarashi, D., Tsuda, K., and Katagiri, F. (2012). The peptide growth factor, phytosulfokine, attenuates pattern-triggered immunity. *The Plant Journal* 71(2)**,** 194-204.
Kampinga, H.H., and Craig, E.A. (2010). The HSP70 chaperone machinery: J proteins as drivers of functional specificity. *Nature reviews Molecular cell biology* 11(8)**,** 579-592.
Kanehisa, M., Furumichi, M.., Tanabe, M., Sato, Y. and Morishima, K. (2017). KEGG: new perspectives on genomes, pathways, diseases and drugs. *Nucleic acids research*. 45 D353-D361.
Katiyar, A., Smita, S., Lenka, S.K., Rajwanshi, R., Chinnusamy, V., and Bansal, K.C. (2012). Genome-wide classification and expression analysis of MYB transcription factor families in rice and Arabidopsis. *BMC genomics* 13(1)**,** 544.
Kim, M.J., Kim, H.R., and Paek, K.-H. (2006). Arabidopsis tonoplast proteins TIP1 and TIP2 interact with the cucumber mosaic virus 1a replication protein. *Journal of general virology* 87(11)**,** 3425-3431.
Lacerda, A., Vasconcelos, É.A.R., Pelegrini, P.B., and Grossi-de-Sa, M.F. (2014). Antifungal defensins and their role in plant defense. *Frontiers in microbiology* 5**,** 116.
Lim, H.-S., Nam, J., Seo, E.-Y., Nam, M., Vaira, A.M., Bae, H., et al. (2014). The coat protein of Alternanthera mosaic virus is the elicitor of a temperature-sensitive systemic necrosis in Nicotiana benthamiana, and interacts with a host boron transporter protein. *Virology* 452**,** 264-278.
Liu, H., Zhou, X., Dong, N., Liu, X., Zhang, H., and Zhang, Z. (2011a). Expression of a wheat MYB gene in transgenic tobacco enhances resistance to Ralstonia solanacearum, and to drought and salt stresses. *Functional & integrative genomics* 11(3)**,** 431-443.

Liu, J.Z., and Whitham, S.A. (2013). Overexpression of a soybean nuclear localized type–III DnaJ domain-containing HSP40 reveals its roles in cell death and disease resistance. *The Plant Journal* 74(1)**,** 110-121.

Liu, Q., Yuan, M., Zhou, Y., Li, X., Xiao, J., and Wang, S. (2011b). A paralog of the MtN3/saliva family recessively confers race-specific resistance to Xanthomonas oryzae in rice. *Plant, cell & environment* 34(11)**,** 1958-1969.

Loivamäki, M., Stührwohldt, N., Deeken, R., Steffens, B., Roitsch, T., Hedrich, R., et al. (2010). A role for PSK signaling in wounding and microbial interactions in Arabidopsis. *Physiologia plantarum* 139(4)**,** 348-357.

Maimbo, M., Ohnishi, K., Hikichi, Y., Yoshioka, H., and Kiba, A. (2007). Induction of a small heat shock protein and its functional roles in Nicotiana plants in the defense response against Ralstonia solanacearum. *Plant Physiology* 145(4)**,** 1588-1599.

Mandadi, K.K., and Scholthof, K.-B.G. (2013). Plant immune responses against viruses: how does a virus cause disease? *The Plant Cell* 25(5)**,** 1489-1505.

Marino, G., and Funk, C. (2012). Matrix metalloproteinases in plants: a brief overview. *Physiologia plantarum* 145(1)**,** 196-202.

Matsumoto, T., Lian, H.-L., Su, W.-A., Tanaka, D., wei Liu, C., Iwasaki, I., et al. (2009). Role of the aquaporin PIP1 subfamily in the chilling tolerance of rice. *Plant and Cell Physiology* 50(2)**,** 216-229.

Meissner, M., Orsini, E., Ruschhaupt, M., Melchinger, A.E., Hincha, D.K., and Heyer, A.G. (2013). Mapping quantitative trait loci for freezing tolerance in a recombinant inbred line population of Arabidopsis thaliana accessions Tenela and C24 reveals REVEILLE1 as negative regulator of cold acclimation. *Plant, cell & environment* 36(7)**,** 1256-1267.

Meng, D., Walsh, M., and Fricke, W. (2016). Rapid changes in root hydraulic conductivity and aquaporin expression in rice (Oryza sativa L.) in response to shoot removal–xylem tension as a possible signal. *Annals of Botany* 118(4)**,** 809-819.

Modolo, L.V., Augusto, O., Almeida, I.M., Pinto-Maglio, C.A., Oliveira, H.C., Seligman, K., et al. (2006). Decreased arginine and nitrite levels in nitrate reductase-deficient Arabidopsis thaliana plants impair nitric oxide synthesis and the hypersensitive response to Pseudomonas syringae. *Plant Science* 171(1)**,** 34-40.

Nagaraj, S., Senthil-Kumar, M., Ramu, V.S., Wang, K., and Mysore, K.S. (2016). Plant ribosomal proteins, RPL12 and RPL19, play a role in nonhost disease resistance against bacterial pathogens. *Frontiers in plant science* 6**,** 1192.

Opperman, C.H., Taylor, C.G., and Conkling, M.A. (1994). Root-knot nematode-directed expression of a plant root-specific gene. *Science-AAAS-Weekly Paper Edition-including Guide to Scientific Information* 263(5144)**,** 221-223.

Park, C.-J., and Seo, Y.-S. (2015). Heat shock proteins: a review of the molecular chaperones for plant immunity. *The plant pathology journal* 31(4)**,** 323.

Paungfoo-Lonhienne, C., Schmidt, S., and Lonhienne, T.G. (2010). Uptake of non-pathogenic E. coli by Arabidopsis induces down-regulation of heat shock proteins. *Plant signaling & behavior* 5(12)**,** 1626-1628.

Rawat, R., Schwartz, J., Jones, M.A., Sairanen, I., Cheng, Y., Andersson, C.R., et al. (2009). REVEILLE1, a Myb-like transcription factor, integrates the circadian clock and auxin pathways. *Proceedings of the National Academy of Sciences* 106(39)**,** 16883-16888.

Rogalski, M., Schöttler, M.A., Thiele, W., Schulze, W.X., and Bock, R. (2008). Rpl33, a nonessential plastid-encoded ribosomal protein in tobacco, is required under cold stress conditions. *The Plant Cell* 20(8)**,** 2221-2237.

Saibo, N.J., Lourenço, T., and Oliveira, M.M. (2009). Transcription factors and regulation of photosynthetic and related metabolism under environmental stresses. *Annals of botany* 103(4)**,** 609-623.

Schiermeyer, A., Hartenstein, H., Mandal, M.K., Otte, B., Wahner, V., and Schillberg, S. (2009). A membrane-bound matrix-metalloproteinase from Nicotiana tabacum cv. BY-2 is induced by bacterial pathogens. *BMC plant biology* 9(1)**,** 83.

Sekhwal, M.K., Li, P., Lam, I., Wang, X., Cloutier, S., and You, F.M. (2015). Disease resistance gene analogs (RGAs) in plants. *International journal of molecular sciences* 16(8)**,** 19248-19290.

Soellick, T.-R., Uhrig, J., Bucher, G., Kellmann, J.-W., and Schreier, P. (2000). The movement protein NSm of tomato spotted wilt tospovirus (TSWV): RNA binding, interaction with the TSWV N protein, and identification of interacting plant proteins. *Proceedings of the National Academy of Sciences* 97(5)**,** 2373-2378.

Song, J., Bradeen, J.M., Naess, S.K., Raasch, J.A., Wielgus, S.M., Haberlach, G.T., et al. (2003). Gene RB cloned from Solanum bulbocastanum confers broad spectrum resistance to potato late blight. *Proceedings of the National Academy of Sciences* 100(16)**,** 9128-9133.

Stevenson, C.E., Burton, N., Costa, M.M., Nath, U., Dixon, R.A., Coen, E.S., et al. (2006). Crystal structure of the MYB domain of the RAD transcription factor from Antirrhinum majus. *Proteins: Structure, Function, and Bioinformatics* 65(4)**,** 1041-1045.

Sun, W., Van Montagu, M., and Verbruggen, N. (2002). Small heat shock proteins and stress tolerance in plants. *Biochimica et Biophysica Acta (BBA)-Gene Structure and Expression* 1577(1)**,** 1-9.

Takano, J., Miwa, K., Yuan, L., von Wirén, N., and Fujiwara, T. (2005). Endocytosis and degradation of BOR1, a boron transporter of Arabidopsis thaliana, regulated by boron availability. *Proceedings of the National Academy of Sciences of the United States of America* 102(34)**,** 12276-12281.

Takano, J., Noguchi, K., Yasumori, M., Kobayashi, M., Gajdos, Z., Miwa, K., et al. (2002). Arabidopsis boron transporter for xylem loading. *Nature* 420(6913)**,** 337-340.

Thomma, B.P., Cammue, B.P., and Thevissen, K. (2002). Plant defensins. *Planta* 216(2)**,** 193-202.

Thomma, B.P., Nürnberger, T., and Joosten, M.H. (2011). Of PAMPs and effectors: the blurred PTI-ETI dichotomy. *The plant cell* 23(1)**,** 4-15.

Tian, S., Wang, X., Li, P., Wang, H., Ji, H., Xie, J., et al. (2016). Plant aquaporin AtPIP1; 4 links apoplastic H2O2 induction to disease immunity pathways. *Plant physiology***,** pp. 01237.02015.

Van Der Vossen, E., Sikkema, A., Hekkert, B.t.L., Gros, J., Stevens, P., Muskens, M., et al. (2003). An ancient R gene from the wild potato species Solanum bulbocastanum confers broad-spectrum resistance to Phytophthora infestans in cultivated potato and tomato. *The Plant Journal* 36(6)**,** 867-882.

Verchot, J. (2012). Cellular chaperones and folding enzymes are vital contributors to membrane bound replication and movement complexes during plant RNA virus infection. *Frontiers in plant science* 3**,** 275.

Wang, J., Hu, Z., Zhao, T., Yang, Y., Chen, T., Yang, M., et al. (2015a). Genome-wide analysis of bHLH transcription factor and involvement in the infection by yellow leaf curl virus in tomato (Solanum lycopersicum). *BMC genomics* 16(1)**,** 39.

Wang, J., Li, H., Han, Z., Zhang, H., Wang, T., Lin, G., et al. (2015b). Allosteric receptor activation by the plant peptide hormone phytosulfokine. *Nature* 525(7568)**,** 265-268.

Wang, Y., Guo, H., Li, H., Zhang, H., and Miao, X. (2012). Identification of transcription factors potential related to brown planthopper resistance in rice via microarray expression profiling. *BMC genomics* 13(1)**,** 687.

Wang, Y., and Wu, W.-H. (2010). Plant sensing and signaling in response to K+-deficiency. *Molecular plant* 3(2)**,** 280-287.

Whitham, S.A., Quan, S., Chang, H.S., Cooper, B., Estes, B., Zhu, T., et al. (2003). Diverse RNA viruses elicit the expression of common sets of genes in susceptible Arabidopsis thaliana plants. *The Plant Journal* 33(2)**,** 271-283.

Xia, Y. (2004). Proteases in pathogenesis and plant defence. *Cellular microbiology* 6(10)**,** 905-913.

Yamamoto-Katou, A., Katou, S., Yoshioka, H., Doke, N., and Kawakita, K. (2006). Nitrate reductase is responsible for elicitin-induced nitric oxide production in Nicotiana benthamiana. *Plant and cell physiology* 47(6)**,** 726-735.

Yuan, H., Chen, X., Zhu, L., and He, G. (2005). Identification of genes responsive to brown planthopper Nilaparvata lugens Stål (Homoptera: Delphacidae) feeding in rice. *Planta* 221(1)**,** 105-112.

Zhou, L., He, H., Liu, R., Han, Q., Shou, H., and Liu, B. (2014). Overexpression of GmAKT2 potassium channel enhances resistance to soybean mosaic virus. *BMC plant biology* 14(1)**,** 154.

Zhou, X., Hao, Q., Liao, J.-m., Zhang, Q., and Lu, H. (2013). Ribosomal protein S14 unties the MDM2–p53 loop upon ribosomal stress. *Oncogene* 32(3)**,** 388-396.

Zipfel, C. (2008). Pattern-recognition receptors in plant innate immunity. *Current opinion in immunology* 20(1)**,** 10-16.

Zou, J., Rodriguez-Zas, S., Aldea, M., Li, M., Zhu, J., Gonzalez, D.O., et al. (2005). Expression profiling soybean response to Pseudomonas syringae reveals new defense-related genes and rapid HR-specific downregulation of photosynthesis. *Molecular Plant-Microbe Interactions* 18(11)**,** 1161-1174.
